# Advanced Formulations Based on Poly(ionic liquid) Materials for Additive Manufacturing

**DOI:** 10.3390/polym14235121

**Published:** 2022-11-24

**Authors:** Sara Miralles-Comins, Marcileia Zanatta, Victor Sans

**Affiliations:** Institute of Advanced Materials (INAM), Universitat Jaume I (UJI), Avenida de Vicent Sos Baynat, s/n, 12071 Castellón de la Plana, Castellón, Spain

**Keywords:** polymeric ionic liquids, 3D printing, advanced materials, catalysis, photoactive, antimicrobial, electronic

## Abstract

Innovation in materials specially formulated for additive manufacturing is of great interest and can generate new opportunities for designing cost-effective smart materials for next-generation devices and engineering applications. Nevertheless, advanced molecular and nanostructured systems are frequently not possible to integrate into 3D printable materials, thus limiting their technological transferability. In some cases, this challenge can be overcome using polymeric macromolecules of ionic nature, such as polymeric ionic liquids (PILs). Due to their tuneability, wide variety in molecular composition, and macromolecular architecture, they show a remarkable ability to stabilize molecular and nanostructured materials. The technology resulting from 3D-printable PIL-based formulations represents an untapped array of potential applications, including optoelectronic, antimicrobial, catalysis, photoactive, conductive, and redox applications.

## 1. Introduction

In recent years, additive manufacturing techniques (AM), also known as three-dimensional printing (3DP), have been intensively researched and are increasingly finding industrial applications as part of Industry 4.0 initiatives [1]. These technologies allow designing and fabricating objects with specific shapes and sizes that would be difficult, and sometimes impossible, with traditional manufacturing techniques. However, the range of available materials, resins, and inks available for AM is limited, thus hindering the growth of the field. The development of formulations tailored specifically for 3DP that can add advanced functionality to the parts fabricated is a promising avenue. In this regard, polymeric macromolecules have been widely employed for printing smart materials, including materials that respond to external stimuli from the environment in a reproducible, reliable, specific, and useful way [2], which is known as 4D printing [3,4,5,6]. For instance, hydrogels are being evaluated as potential materials, since they are excellent candidates for biomedical applications given their biocompatibility [7,8,9]. Furthermore, emerging polymers with greatly varied chemical structures and properties have been developed for AM, including covalent organic frameworks (COFs) [10], polypeptides [11], ionogels [12,13,14], and many more [15]. Recently, polymeric ionic liquids (PILs) have gained prominence as intelligent materials [16] capable of responding to temperature [17], light [18], solvents [19], pH [20], and CO_2_ [21]. Ionic liquids (ILs) and polymeric analogues are a fascinating field of research, due to the possibility of controlling their macroscopic properties by changing the formulation (cation/anion) [22,23]. These polymers can be specially designed to be printed by 3D techniques allowing the design and manufacture of the high-resolution multi-material devices essential for the development of future technologies [24].

This review emphasizes the state of the art of 3D-printable PIL-based materials and their most common applications, as found in the literature. The countless cation–anion combinations (it is theoretically feasible to generate 10^18^ distinct ILs) possess unique physicochemical properties that can be tuned easily, without sacrificing 3D printability and resolution [25,26]. Moreover, PILs are ideal matrices to stabilize molecular and nanostructured materials, due to their ionic and supramolecular interactions. These polymeric compounds can encapsulate smart molecules, directly translating their advanced properties to the macro scale through additive manufacturing [27]. The development of printable PILs is an expanding area, although it is still in its infancy.

**Figure 1:**
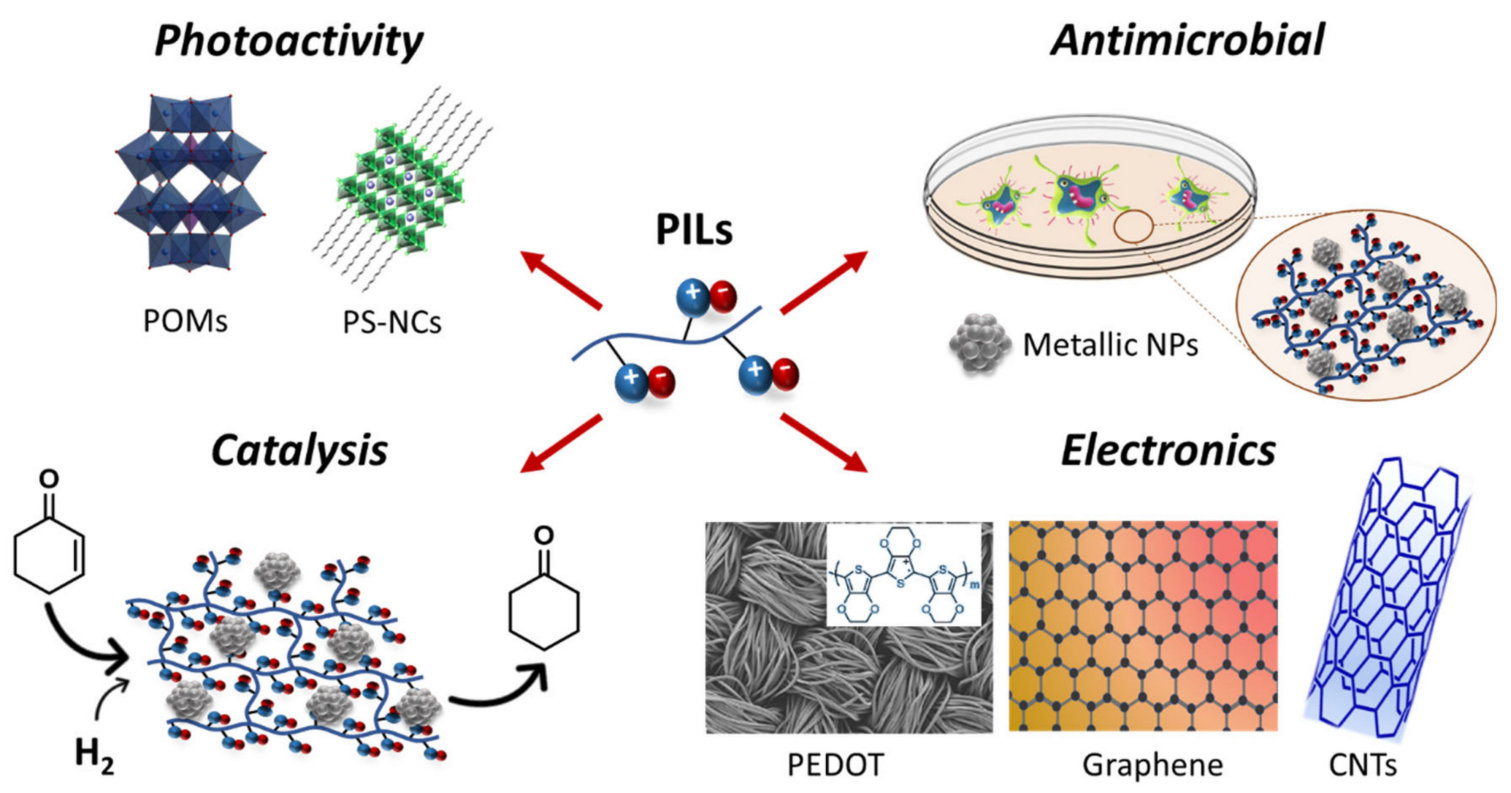
Common applications of 3D printable PILs capable of stabilizing active materials. (PEDOT: Adapted from Du et al. [28] (under CC BY-NC-SA 4.0 license)).

First, the different AM techniques are introduced, as well as the unique features of ILs and PILs. Next, we highlight the most relevant applications of 3D-printed PILs that have been reported. A wide variety of advanced molecular materials (AMMs), such as perovskites nanocrystals (PS-NCs), polyoxometalates (POMs), and metallic nanoparticles (NPs), as well as carbon-based materials and conductive fillers, have been embedded into PILs to enhance the performance of printed devices in different applications (Figure 1). Owing to the variety of chemistries and systems included in this analysis, each example is explained briefly and the reader is referred to the original literature for further information. Finally, we provide a summary of the current challenges and future perspectives of 3DP. The advances in polymer science and AM technologies needs to be maintained, in order to create next-generation devices. There is a tremendous opportunity for novel materials to forge new paths toward applications that had not previously been considered because AM was unavailable.

## 2. 3D Printing Overview

The recent growth of AM techniques is attributed to several advantages, including the rapid translation of fundamental molecular properties, fabrication of complex geometries with high precision and resolution, unprecedent flexibility in design, waste minimization, and personal customization, which enables the fine control of structural features [29]. Furthermore, it enables distributed production, as opposed to the logistically complex production lines with conventional production methods. Whereas traditional fabrication techniques have limitations in controlling the geometry and architecture of macroscopic components, without compromising the performance, 3DP techniques are an efficient strategy to engineer well-controlled functional materials across scales (from nano to macroscale) with accurate control over the geometry (dimension, porosity, and morphology) and structure. Moreover, 3DP expands the boundaries of materials science and provides an exciting opportunity for interdisciplinary research, as it allows the incorporation of multiple nano-materials in the same printing process, to obtain multi-functional devices. 

The main feature of AM is to design an object by adding material in a layer-by-layer method until the model is completed. The first step is to generate data from a three-dimensional computer-aided design (3D CAD) software, in a simple machine avoiding complicate production chains or process planning. Then, the digital CAD model is translated into computer readable format as an STL file (an industry-standard stereo-lithography format), which slices cross-sections into codes that allow the machine to build the object before printing. This is a fast, reliable, and facile way to design prototypes and devices, and additionally, this 3D CAD file can be easily shared and modified, with large online databases of designs shared under creative commons licenses [30].

Additive manufacturing is a booming area. According to the Smithers Group—a provider of strategic market research reports—billions of dollars are invested in the 3DP market and annual growth is expected until 2027, potentially reaching $55.8 billion. Nevertheless, in 2021, the AM industry growth was not as high as expected (growing only 7.5%, which was quite low compared to the average growth of 27.4% observed over the previous 10 years), due to the pandemic situation that affected all countries [31]. 

Additionally, quantitative life cycle analysis (LCA) data shows that the process of AM reduces environmental impacts compared to other existing manufacturing technologies. Indeed, the additive nature of these technique minimizes the amount of material employed, and reduces the energy required and the resources demanded. Furthermore, it is estimated that the implementation of printing processes might lower carbon dioxide emission intensities by about 5% by 2025 [32]. 

The first rapid prototyping system, “stereolithography apparatus” (SLA), was patented by Charles Hull in 1986 [33]. Since then, many different types of AM techniques have been developed and commercialized. Depending on the form of the starting material and the way the materials are deposited and assembled in the additive fashion, 3DP techniques can be divided into seven categories, opening new pathways for creating devices with outstanding performance at fine resolutions [34]. These techniques cover a broad range of scales, from micro- to macroscale devices, and materials (Table 1). All of them have been widely studied in various literature reviews, in which the materials, benefits, drawbacks, and main application areas of each printing method were specified [35,36,37,38]. According to the technique employed, the parts may be post-processed to improve their strength or surface quality. This work mainly focuses on vat polymerization, as further explained above.

Over recent years, different methods such as Inkjet [40] and FFF [41] have been employed for 3D manufacturing of PIL materials; however, vat polymerization has become the preferred technique for printing PILs. Both the DLP [42,43] and SLA [42,43] methods have been widely used in this area, for this reason VP techniques will be reviewed in more detail.

### 2.1. Vat Polymerization Techniques

The vat polymerization method is so called because it is carried out in a vat containing a liquid photopolymer that is selectively irradiated by UV light. The principle of this technology is based on the chemical reaction known as photopolymerization. This process takes place when liquid light sensitive inks, composed of monomers, photo-initiator, additives, and fillers are polymerized or cured upon light irradiation. As the reaction proceeds, the system’s molecular weight increases, and so does the degree of branching if the formulation contains cross-linkers. Depending on the type and number of reactive groups in the monomers/oligomers involved, different macromolecular structures can be synthesized: linear, branched, or cross-linked [44].

During the 3DP process, the object is built on a platform that moves up and down, while a sufficiently strong light source irradiates each layer, in order to activate the photopolymerization reaction. This step is repeated layer by layer, by immersing the polymer parts into the liquid monomer each time. Therefore, it is critical that the solid parts are insoluble in the liquid resin. The printing does not require complex machine configurations and supporting structural materials, and the final macroscopic models are solids that can be used immediately. However, a post-printing treatment is usually employed, to remove the excess of uncured resins, as well as a photo-curing or heating process to improve the mechanical properties [45].

**Figure 2:**
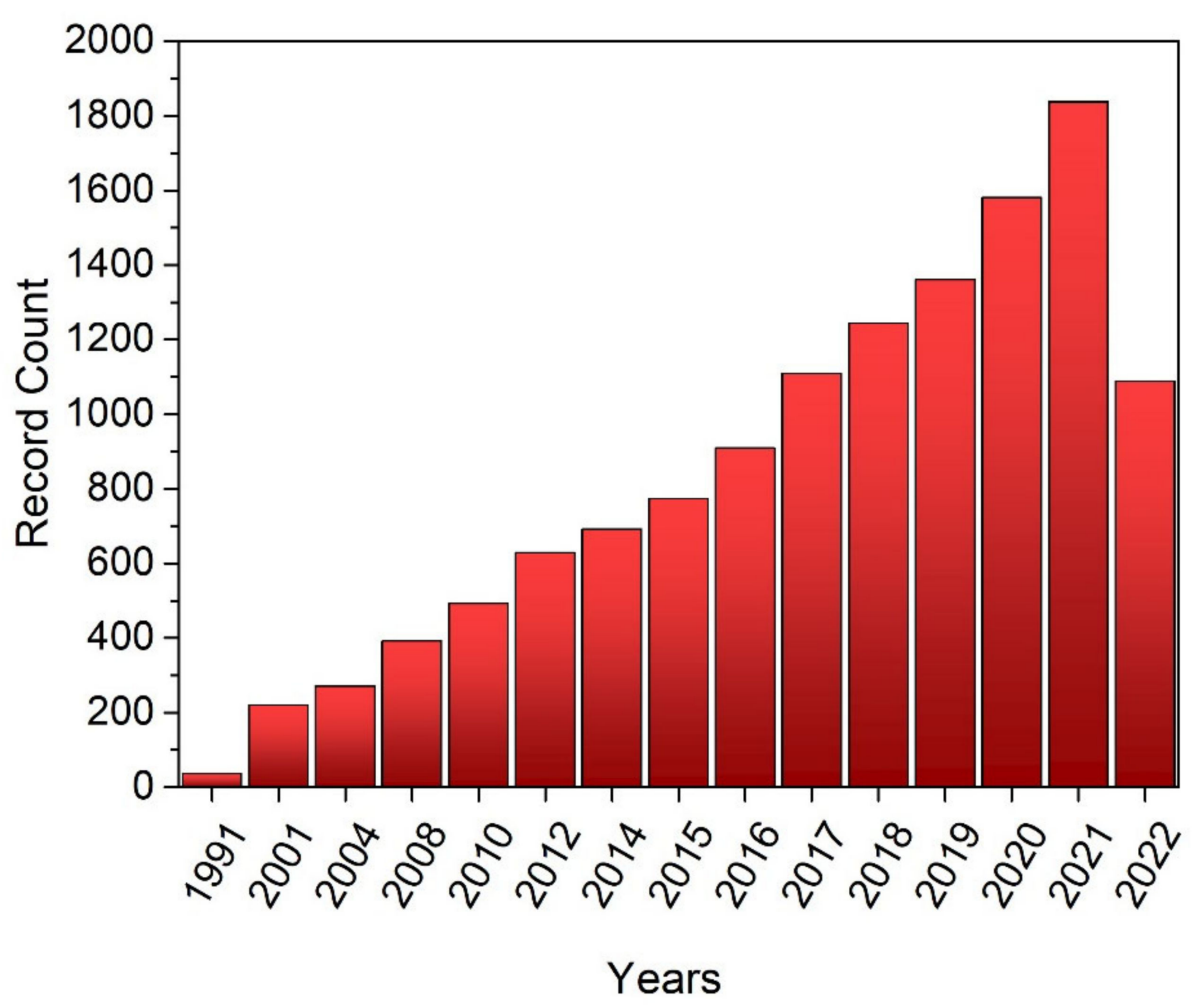
Publications on VP in the last three decades. Data from Web of Science database.

This technology was developed in the 1980s, being the first patented AM system. However, in recent years, interest in this methodology has been growing, both in the industrial and research fields. Photopolymerization techniques are gaining importance due to the flexibility in the development of starting printable materials that can be used, opening a broad range of choices. Furthermore, complex-shaped and customized structures can be produced quite quickly (depending on the size), with excellent printing resolution [46]. This trend is reflected in the increasing number of scientific articles involving vat polymerization over the last decade (Figure 2). 

**Scheme 1:**
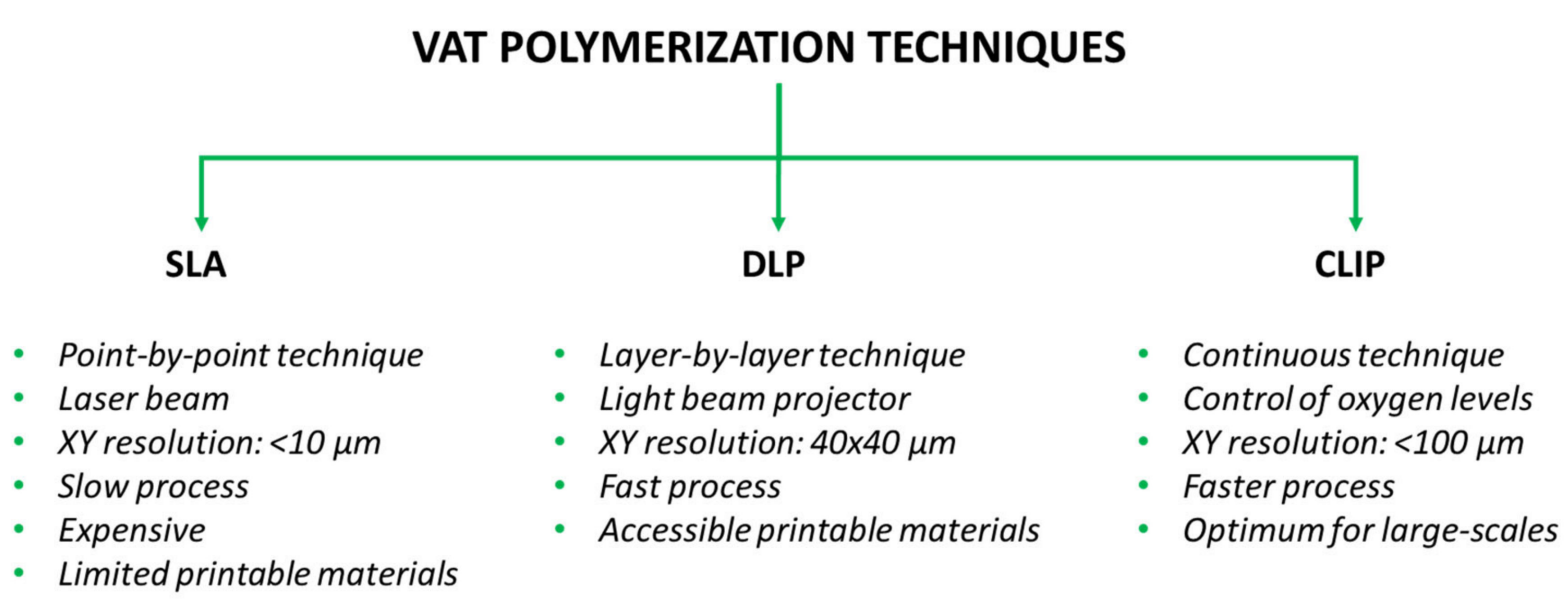
Main characteristics of the different VP methodologies.

Within VP technologies, there are three main techniques, as summarized in Scheme 1: stereolithography (SLA), digital light processing (DLP), and continuous liquid interface production (CLIP).

(a) Stereolithography (SLA):

SLA is a well-established AM technology, since it was one of the earliest methods implemented. This technique utilizes a laser beam that draws the customized design on the photopolymer surface and selectively cures the liquid resin contained in the vat point-by-point (X–Y plane). SLA is mostly used to create high-quality objects, since it can achieve features <10 μm at a fine resolution. However, it is slower than other AM techniques, quite expensive, and the range of printable materials is very limited [29,35]. A derivate of SLA technology has also been reported called micro-stereolithography 3D printing (μ-SLA or MSLA), which can achieve 5 μm as a minimum feature size [47].

(b) Digital light processing (DLP):

This technique involves the irradiation of an entire layer simultaneously using a light beam projector. The substitution of the laser by a beam allows building parts much more quickly than SLA [48]. Although laser technology presents higher printing resolutions with finer results, in terms of surface finishing [46], commercial DLP technology is able to reach a layer thickness of down to 5 μm and resolutions as small as 40 × 40 μm [49]. In optimized research setups, features of 18 × 20 μm for 3D serpentine flow channels were reported using a custom digital light processor stereolithographic (DLP-SLA) 3D printer and a custom resin [50].

(c) Continuous liquid interface production (CLIP):

An innovative variation of DLP-3D printing is the CLIP method, which uses a projector that works continuously, instead of layer-by-layer, by controlling oxygen levels throughout an oxygen-permeable membrane. The printing speed is increased dramatically, being the fastest VP technique, but the accuracy obtained is not as high as with DLP, with feature resolutions of less than 100 μm [51,52]. Large-scale structures can be produced with the CLIP approach [53].

In addition to these, there are other types of photopolymer-based 3DP that have been developed in recent years; for example, two-photon polymerization (2PP) and CAL (computed axial lithography); however, they suffer from some limitations, such as a slow printing speed and expensive equipment [54]. In 2PP, the photo-initiator absorbs two photons simultaneously [55,56]. Additionally, the laser point can be moved in the X–Y–Z directions, to polymerize the resin, making it possible to use high-viscosity photopolymers and create complex geometries without supporting structures [57]. CAL is based on the computed tomography (CT) scans technique to generate a hologram [58]. The hologram is created by simultaneously projecting multiple 2D images that propagate through the resin from different angles as the vat container rotates, generating a 3D image with sufficient energy for light curing [59].

### 2.2. Photopolymers

Manufacturing materials with advanced properties specifically designed for 3D printing are highly desirable for a variety of applications, ranging from household goods, personalized healthcare, tissue engineering, and medical devices, to renewable energy, automotive components, and soft robotics. The loop between design, manufacture, and application can be rapidly closed with AM. Hence, the unique properties of molecular and nanostructured materials can be directly transferred to macroscopic functional devices. The possibility of integrating advanced molecules into 3D printable materials, while simultaneously controlling their geometry, is a great contribution and will accelerate the development of novel devices with complex 3D geometries. In this way, printing technologies can generate smart materials with specific characteristics using the appropriate inks [60,61]. 

**Figure 3:**
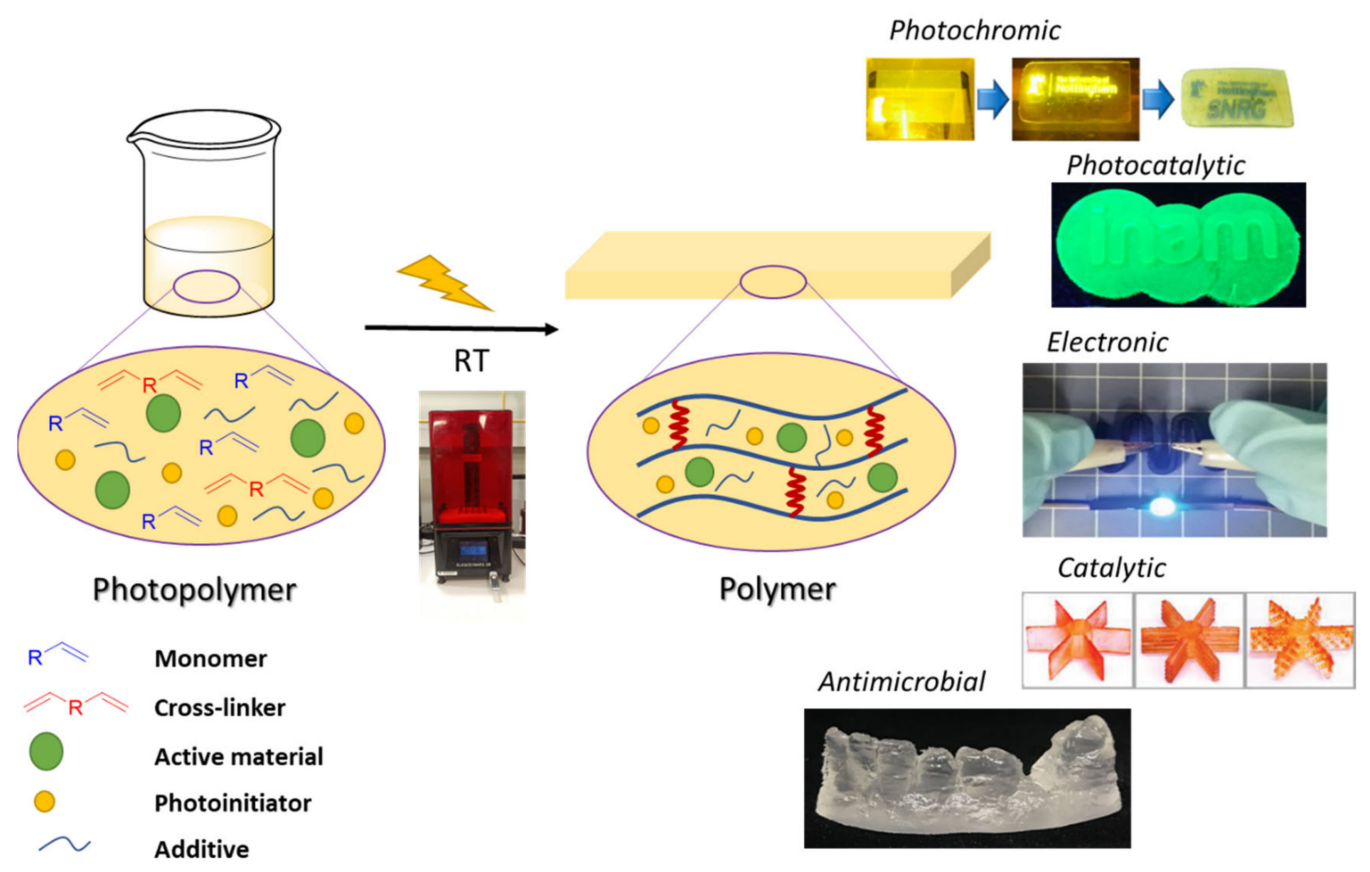
Representation of the vat photopolymerization process and some demonstrated applications. Adapted from references [26,27,65,66].

In particular, photopolymers have attracted much attention as printable resins, due to their ability to stabilize nano and molecular composites, their low toxicity, wide variety of materials, and facility of tuning chemical composition and architecture [3]. The photopolymer is the liquid precursor of the polymer and can be polymerized by several methods, although light-induced polymerization offers remarkable advantages. It delivers rapid polymerization rates, room temperature treatments, low energy requirements, the use of solvent-free formulations, a broad range of wavelengths (from X-rays to NIR) to activate the photopolymerization [62,63], and a high dimensional accuracy, as the photopolymerization can be controlled by managing the light settings during the process. Despite all these positive qualities, the main benefit of photopolymers is that the resin can be tailored to meet precise requirements [29]. The combination of the appropriate elements (monomers, active materials, photo-initiators, and additives) must be fine-tuned to achieve specific properties (e.g., chemical, mechanical, optical, etc.) and the desired resolution [45,54]. In conclusion, the photopolymer can be custom-prepared by combining chemical and reactive compounds (both liquid and powder) to meet the intended application (Figure 3) [46]. Some recent studies have shown great progress in employing self-assembling molecular systems with stimuli-responsive behavior to specific external stimuli, providing new functionalities to additively manufactured objects [64]. The type and degree of stimulus response of smart polymers can be regulated through precise synthetic design or via incorporation of additives [4,5,6].

Photopolymers are widely used in vat polymerization AM techniques and also in inkjet, multijet, and some binder jetting methods. The only important issue to take into account is that both the digital design (3D CAD model) and the printing parameters must be properly optimized considering the photopolymer composition, in order to obtain high-resolved 3D-printed structures [67]. A typical problem that emerges while preparing a photosensitive mixture is the low affinity of the components used, which has a direct influence on the printed formulation’s homogeneity if they are not effectively integrated [68]. For instance, most photopolymer resins are non-polar reagents, while other components, such as dyes, have a different polarity [69]. Mixing those components directly might cause solvation problems that create light scattering or reflection during the 3D printing step due to the dye’s aggregation. Thus, inhomogeneous baselines will lead to low resolutions and inaccurate printings, which could negatively affect the final application [70]. Using a suitable reactive diluent (compatible with the polymeric matrix) as an intermediate reagent is a way to boost the additive solubility in the monomer. This issue can be also found when adding some nanostructured active materials, in order to enhance specific characteristics (such as the conductivity or the mechanical strength) of the printed objects [71]. The addition of such materials can change the matrix properties, including the transparency [72] or viscosity [73], which may affect the resolution of the printed parts. Similarly, the incident light that activates the polymerization reaction can be modified by the additive, since it can also absorb part of the light [74]. Therefore, it is important to find a balance between the amount of nanomaterial and photopolymer printability, in order to design printed parts with the appropriate form and qualities [75]. In some cases, a promising solution to avoid printing constraints, without compromising the quantity of active material added to the resin formulation, may be to functionalize the active materials, to increase their compatibility with the rest of the resin formulation, thus favoring solubility, dispersibility, and stability [73,76,77]. Overall, there is a need to explore and broaden the availability of functional printable materials or, alternatively, materials capable of stabilizing and properly integrating smart agents in homogeneous and compatible formulations.

One of the avenues that is currently gaining popularity for developing novel materials is exploration of the structure–property relationship of ILs, given their limitless tunability through changing the corresponding ions. ILs and PILs have received increased attention for developing next-generation 3D printed complexes, due to their unique properties, such as their extended ion-conductivity and enhanced charge stability [24]. Polymeric resins and ceramics are mainly electrically insulating, while some metals may be chemically unstable, requiring long post-treatments that might not be compatible with all components in the formulation [34]. Normally, conductive materials are manufactured by conventional methods using conductive composites, which are expensive, require extra energy, and often lead to composite leaching. Printable IL materials are opening new opportunities in material design, as well as broadening applications for 3D printing technologies. For example, Long and co-workers prepared a PIL-based 3D printable resin that was much more conductive, stable, and environmentally friendly than using styrene, a common neutral polymer [78]. In a short period, these materials have had a significant impact across various areas of AM (e.g., photoactive, antimicrobial, catalyst, electronic, robotic, and bio applications) [24,41,66,79,80,81].

## 3. Polymeric Ionic Liquids

### 3.1. Properties of Ionic Liquids

ILs are organic molten salts in which the ions are poorly coordinated and the salts melt below 100 °C, or even at room temperature. The first IL, ethanol ammonium nitrate, was reported in 1888 [82], but it was few decades ago when the vast potential of these compounds was noticed. The renewed interest in them was due to their unique characteristics: chemical and thermal stability, high viscosity, wide electrochemical windows, low vapor pressure, specific solvating ability, good tunable solubility, wide solid to liquid range, and high ionic conductivity. The number of publications related to ILs has been increasing sharply every year for the last two decades, indicating the relevance of these materials [83,84,85]. Initially, these materials were mainly used as solvents for organic synthesis and catalysis, since they were more desirable than conventional volatile solvents [86,87,88]. Indeed, due to their low flammability, negligible vapor pressure, low melting point, and non-volatility properties, ILs were considered “green solvents” [89,90], although some concerns about their toxicity have appeared [91,92,93]. Nevertheless, these ionic compounds can contribute to making chemical production and usage more sustainable [94]. 

Furthermore, they can create a biphasic system, having the catalyst solubilized, while the products are insoluble in the IL [95,96], a feature that has also been widely used in analytical chemistry for separation procedures [97]. Progressively, the uses of ILs have broadened, being used in CO_2_ capture and separation systems [98], in electrocatalysis [99,100,101,102], in pharmaceutical industries [103], and even in space technology [104]. In particular, these chemicals have shown a lot of promise in the development of green catalytic technologies [105]. ILs can play the role of solvent in catalytic reactions, since they are able to dissolve a wide range of organometallic and inorganic compounds. Furthermore, the ionic behavior of these materials can stabilize the catalytic species and intermediates [106,107]. Moreover, the ILs themselves have been used as the catalyst in a variety of reactions, from biodiesel production, to CO_2_ conversion [108,109,110]. In some studies, ILs acted as both a solvent and a catalyst simultaneously [111]. Likewise, they have been extensively used in electrochemical applications as their charge density is much higher than that of a traditional salt solution [112,113]. Electrolytes based on ILs offer several benefits over aqueous and organic electrolytes, making them attractive for use in flexible and stretchable energy storage systems [114,115,116,117,118], high temperature supercapacitors [119,120,121], lithium-ion batteries [122,123], and electrochemical CO_2_ reductions [124,125].

Doping IL into polymers is another process that has recently been investigated, yielding significant changes in both conductivity and overall mechanical properties [126,127]. Pure polymer electrolytes generally have a limited ionic conductivity, thus adding an ionic moisture can be very beneficial [128,129]. The addition of ILs can benefit, not only neutral polymers, but also conductive polymers [130,131].

**Figure 4:**
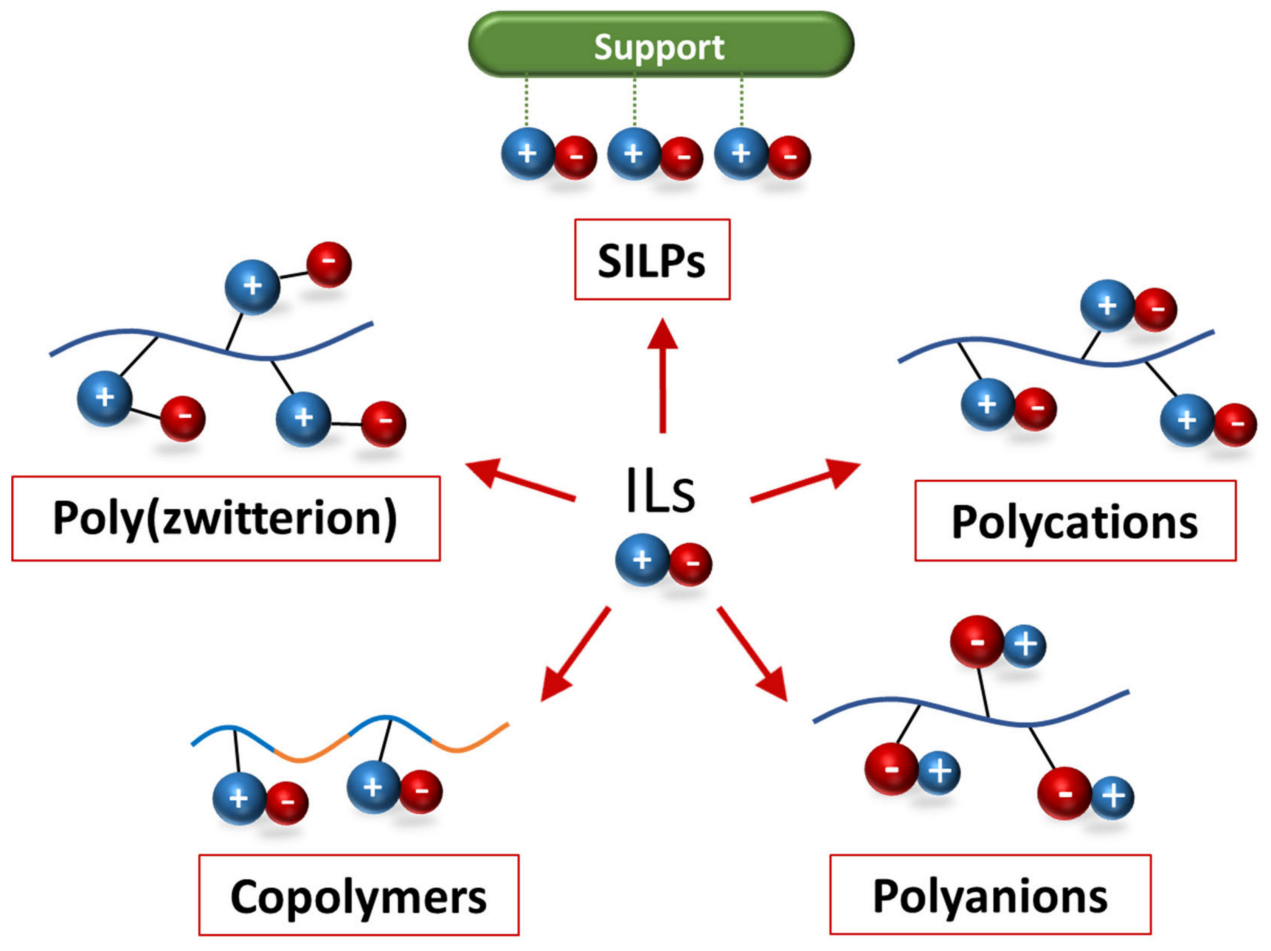
Schematic representation of polymeric materials produced from ILs.

Despite ILs being very popular in both the academic community and industry, due to their well-known advantages [132], some downsides have been highlighted. The main problematics in using ILs are related to processing difficulties. For example, in catalysis or as a solvent the employment of ionic moisture can complicate the purification procedure of the product, and it is almost impossible to recycle them. Moreover, ILs cannot be used in fixed bed reactors, which, along with certain toxicity concerns, are critical challenges to overcome for future scale-up. Additionally, these compounds are liquids, which means that they can liquify and leach over time. An initial approach to overcoming these disadvantages was to use supported ionic liquid phases (SILPs) prepared by immobilizing the ILs onto solid supports [133,134,135]. In many cases the performance was improved, since the properties of ILs were transferred to the solid support, and simultaneously working with a solid material offered additional benefits [135,136,137]. Further methods have been developed to prepare IL-based polymers such as in situ polymerization of neutral vinyl monomers with non-polymerizable ILs or directly polymerizing an ionic monomer of IL structure [138,139,140,141]. A variety of polymeric IL systems, including polycation-type ILs [141], polyanion-type ILs [142], copolymer [139], and poly(zwitterion) [141], have been reported, as summarized in Figure 4.

### 3.2. Properties of Polymeric Ionic Liquids

**Figure 5:**
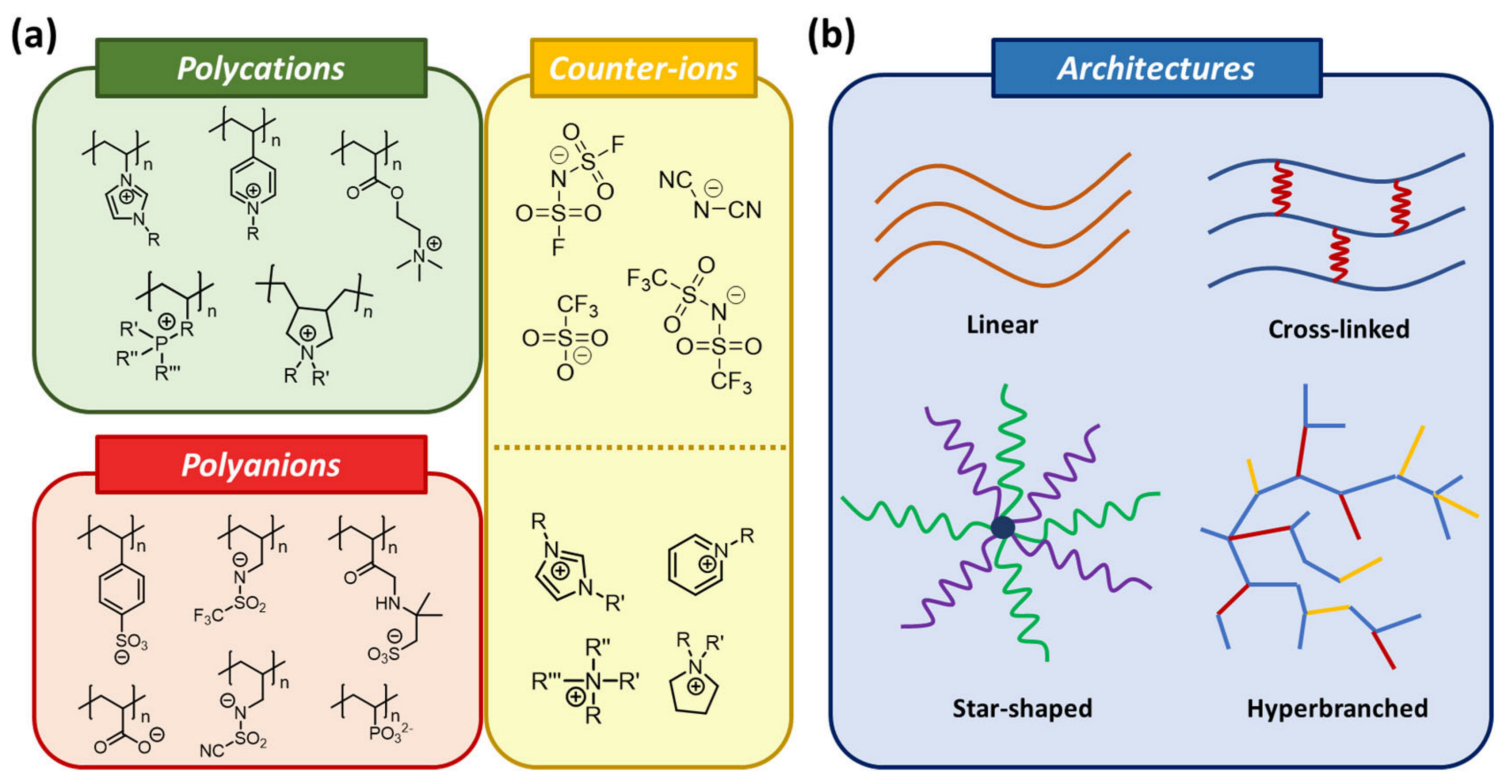
(**a**) Most common structures of polycations, polyanions, and counter-anions of PILs; (**b**) Different polymeric architectures.

Polymeric ionic liquids/poly(ionic liquids) (PILs) are the union of multiple anionic or cationic monomers, forming a polymer backbone with its counterion species bonded to it. These high-molecular-mass compounds combine attractive IL properties with polymer properties (i.e., durability, low toxicity, and easy polymer processing). In other words, during the development of PILs, it is possible to preserve the specific features of ILs and improve other ones by taking advantage of the polymer’s nature, including mechanical robustness, thermal stability, hydrophilicity, rapid manipulation, optimum assembly, and stabilizing properties [143]. Another unique feature of PILs is that the properties of the polymers can be controlled by changing the corresponding ions. Depending on the ion pairs it is possible to vary the polymer’s solubility, fusibility, hydrophilicity, ionic conductivity, molecular mass, glass transition temperature, heat resistance, and thermal stability in a wide range. Most PILs are the nitrogen-based polycationic type, namely imidazolium, pyridine, pyridinium, quaternary ammonium, and cholinium; although phosphorous-containing cationic PILs are gaining popularity (Figure 5a). While ILs can only undergo structural variation through ion metathesis or cation/anion exchange, PILs can be modified by selecting cations, anions, side chains, and functionalities. Even when using the same ionic species and the same number of monomers, different PILs can be built by only changing the architecture: linear, hyperbranched, or star-shaped polymers; obtaining an unlimited library of species (Figure 5b) [144]. Homopolymerization is an important route to prepare PILs; however, other types of polymers can be added covalently to the PIL to form PIL block copolymers.

In an initial approach, PILs were considered for improving the properties of ILs. The tailorable physicochemical properties of PILs have provided the basis for the further design of smart materials, implementing this new class of polyelectrolytes in fields from material science and chemistry to medicine; with applications related to energy and environment, analytical chemistry, electrochemistry, biotechnology, bioelectronics, catalysis, and surface science, among others. Thus, these multifunctional polymers have been recognized as a separate class from ILs, and extensive work has been done in this direction [144,145,146]. 

Solid electrolytes are very desirable in many electrochemical devices, in order to avoid leakage issues and, additionally, they can improve certain electrochemical properties, such as energy density, rate capability, and cycling stability. In this regard, PILs are often less fragile and less hygroscopic than traditional polyelectrolytes. Moreover, electrochromic devices and actuators using PIL-based electrolytes have a better performance, cyclability, speed, and long-term stability [22,147]. The ionic polymers are both permanent and strong polyelectrolytes owing to the IL species’ solvent-independent ionization state. The unique dielectric properties of PILs makes them potentially useful, not only as polymer electrolytes, but also as sorbents and dispersing agents [148,149]. Several groups have investigated PIL-based electrolyte membranes for use in alkaline fuel-cell operations, where the anion exchange membrane acts as an electrolyte to transport anions from the cathode to anode and is one of the key components of alkaline fuel cells [150,151,152]. The use of PILs as binders for electrodes and/or electrolytes in the design of solid-state supercapacitors is also remarkable [153,154]. The addition of PILs into graphene electrodes, for instance, improves the contact between the conductive carbon and the liquid electrolyte, facilitating the diffusion of ions into the electrode and increasing the specific capacity of the supercapacitor by 130% [155]. Moreover, the addition of PIL improved the cyclability of a supercapacitor, keeping the specific capacitance almost unaltered after multiple cycles.

In addition, PILs are ideal materials to stabilize and solubilize molecular and nanostructured materials, as a result of their ionic and supramolecular interactions; hence, these polymeric compounds are extensively used to stabilize smart materials [156,157]. In the fabrication of composite materials using light-based 3DP techniques, it is crucial to avoid phase separations, to avoid the presence of turbidity that will undesirably scatter light and thus reduce the printed resolution. Generally speaking, ionic polymers are able to homogeneously distribute a broad range of additives in their matrices, thus minimizing these problems. For example, some reports demonstrated the excellent capabilities of PILs to immobilize and stabilize metal nanoparticles, and acting as active catalysts in heterogeneous catalytic reactions [158,159,160]. The nanoparticles can even be synthesized and immobilized simultaneously, forming the nanoparticles in situ during the polymerization process [161]. This approach is not limited to metallic nanoparticles, as a broad range of nanoparticles [162,163,164,165], macrostructures such as polymers [166,167,168,169], carbon-based materials [170,171], and enzymes [172,173,174] have been successfully stabilized or immobilized by PILs. A wide variety of functional materials, including porosity-switchable membranes and coatings, [175] thermally responsive compounds [176], biosensors [177], flexible sensors [178], and reversible systems that switch hydrophobicity/hydrophilicity by exchanging the anion [175] and the cation [179], have been explored.

Nevertheless, it is worth remembering that PILs have one of their charges polymerized, becoming a single-ion conductor. Therefore, the conductivity of neat PILs described in the literature up to now has usually been low and is still insufficient for practical use, due to the reduction of the ion mobility. Another factor that has a direct influence on conductivity is the increase of the glass transition temperature after polymerizing. Several times, it has been found that the ionic conductivity values of PILs are at least two magnitudes lower than the corresponding IL monomer unit [135]. Accordingly, the conductivity of PILs needs to be improved by doping with other materials such as ILs, metallic nanoparticles, conductive polymers, AMMs, etc.; taking advantage of the good stabilization properties of PILs. The preparation of mixtures of ILs with PILs has been explored for different applications [153,154,180,181,182]. In this context, different ILs and PILs have been combined successfully without observing segregation into two phases, due to the similarity in their chemical structure. Completely compatible formulations have been used, for instance, as polymer electrolytes, enhancing their performance and conductivity compared to conventional polyelectrolytes [183,184], improving the device lifetimes [185], the solar cell efficiency [186], and the electromechanical performance of the actuators [187]. By mixing IL and PIL in different ratios, the electrolyte can be tailor-made. The ionic conductivity varies significantly (from 10^−5^ S/cm to 10^−2^ S/cm) after increasing the IL content. In addition, the physical appearance of the polymer electrolytes changes from a transparent solid film (100% PIL) to a very viscous and sticky gel (low PIL/IL ratio), with intermediate states, where the polymer electrolytes are usually rubbery and transparent [22,185]. 

Another matter to be considered when using PILs and ILs is that the properties of these materials can be varied greatly just by changing the ions. This is helpful for designing customized PILs. However, in order to meet specific requirements, it is necessary to have a clear understanding of how chemical differences affect the final features. Depending on the chosen PIL, important properties such as the thermal stability can vary from 150 to >400 °C [22,188], measured as the onset of thermal degradation by TGA, and its electrochemical window from 2.5 to 5.0 V [189]. Solubility provides clear evidence of the variation across PILs. For instance, poly(1-vinyl-3-alkyl-imidazolium) with bromide anions is soluble in water as a conventional polyelectrolyte and, after substituting the halide anion with tetrafluoroborate or hexafluorophosphate, the polymer becomes soluble in methanol and polar aprotic solvents, but no longer in water. Then, changing the counter-anion for a fluorinated bis(trifluoromethanesulfonamide) anion or an hydrophobic anion, the polymer become soluble in non-polar solvents [23]. It is therefore essential to know all the characteristics of each PIL or IL before using them.

In conclusion, to obtain the best performance from PILs, it is imperative to combine PILs with advanced materials, to produce smart composites. The ionic nature of PILs ensures their outstanding compatibility with other compounds, since the active ions across the polymer chains can promote interactions with other reactives [156]. The hybrid materials obtained after those unions are notable for having an enhanced performance, such as increased electrical conductivity, thermal stability, antimicrobial activity, and optical properties [155]. Moreover, PILs can be manufactured using 3D methods, which makes them promising candidates for developing next-generation devices. For all these reasons, 3D printable PIL nanocomposites are an exciting and promising research field.

In the following section, we will focus on the reported applications based on printable PILs mixed with AMMs, conductive polymers, and carbon-based materials (carbon nanotubes (CNTs), graphene, graphite), among others.

## 4. Applications of 3D-Printable PILs

At present, the development of polymers especially designed to be printed using 3D techniques is of huge interest. At the same time, in both academic research and industry, there is a need to create polymeric systems with controlled precision in their architecture, domain size, functionality, polarity, solubility, and reactivity. In this context, poly(ionic liquids) combine the tunable properties of ILs with the intrinsic features of polymers, becoming an invaluable material for additive manufacturing. However, the employment of polymerizable ILs as inks for AM techniques is an underexploited area of research.

**Figure 6:**
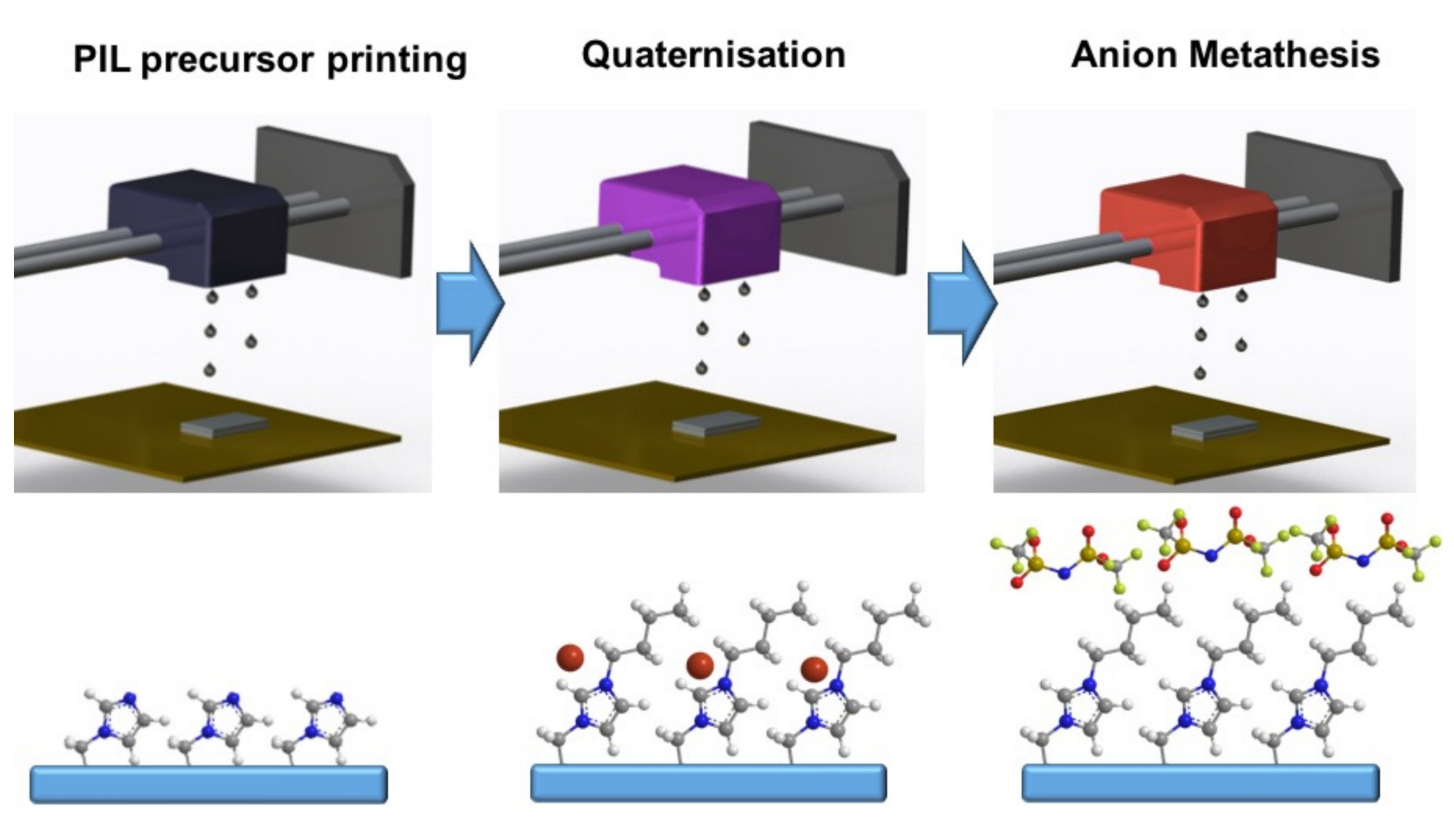
Schematic representation of the method proposed for inkjet printing polyionic films. Reprinted and adapted with permission from reference [40]. Copyright 2018 American Chemical Society.

In 2014, the first study using printable ionic polymers was reported. 4-vinylbenzyl trioctyl phosphonium bis(trifluoromethanesulfonate)imide mixed with diacrylates was photopolymerized by mask projection micro-stereolithography with a low UV light intensity and high digital resolution. The IL monomers were polymerized during the printing process together with other comonomers, in the presence of a cross-linker and a photo-initiator. The resulting electro-active membranes showed high thermal stability, optical clarity, and ion-conductivity [42]. In that example, the IL monomers were viscous, hence the formulation was not appropriate for all printing techniques. A totally different strategy was proposed for an inkjet method, generating a variety of low viscosity printable polycationic materials with finely tuned mechanical, thermal, and superficial properties. The post-polymerization and modification of the polymer allowed improving the mechanical properties of the printed parts (Figure 6) [40].

On the other hand, an important trend in the development of new materials is towards being lightweight, ultrathin, and flexible; the combination of different materials to obtain superior properties in multifunctional devices being necessary. Some required features, including higher stability, durability, catalytic activity, and a lower cost, are crucial for new types of materials used in medical, electronic, and environmental fields. In this context, smart materials such as nanomaterials and carbon-based materials are promising alternatives to meet these requirements, since they can improve the mechanical and electrical properties of polymer matrices. Over the past 20 years, a tremendous expansion in the development of nanostructured composite systems has emerged and been employed in a wide range of nanomaterial applications [190].

### 4.1. Photoactive

**Figure 7:**
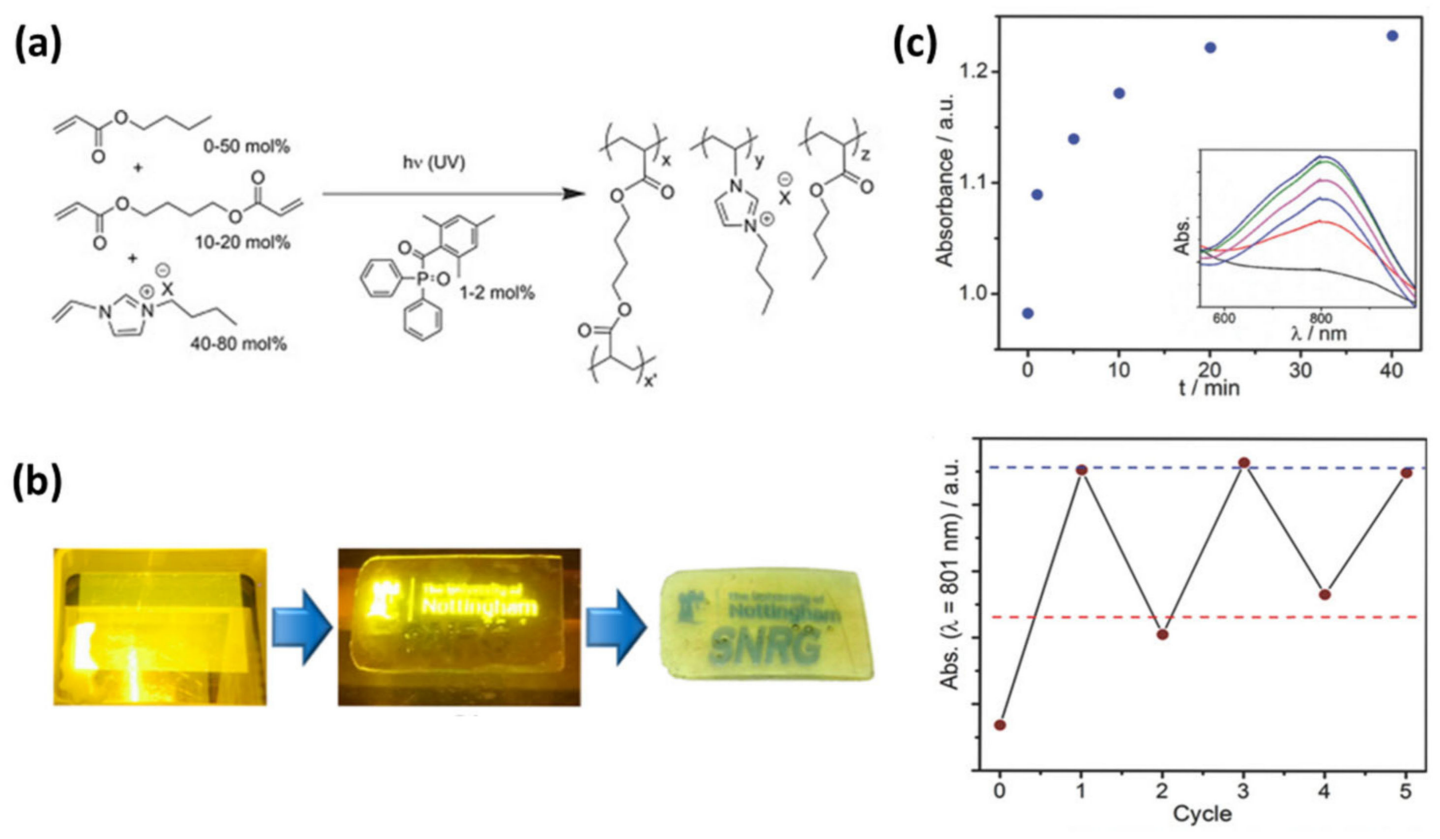
(**a**) Compositions of the PIL-based 3D printing formulation capable of stabilizing the POMs (C_10_POM@[BVIM][NTf_2_]) that were introduced before the printing process. The most significant results were obtained with the C_10_POM@[BVIM][NTf_2_] materials, formed by the combination of the decyloxy(phenylphosphonato-) bifunctionalized POM, (C_10_POM) with 3-butyl-1-vinylimidazolium bis(trifluoromethane)sulfonimide ([BVIM][NTf_2_]); (**b**) Controlled topological and temporal photoreduction of the POM material with visible light to encode information; (**c**) (**top**) Increase in absorption at λmax = 801 nm due to photoreduction of the encapsulated POMs upon exposure to the project light over time, with the corresponding UV–vis spectra inset; (**bottom**) Absorbance intensity at λmax = 801 nm upon cycles of photoreduction (encoding) and oxidation (erasure). Adapted from reference [27], with permission.

Our group has demonstrated, for the first time, the possibility of successfully designing functional advanced multi-materials and devices based on PILs with embedded hybrid organic–inorganic polyoxometalates (POMs). POMs are self-assembled large mixed metal oxides, typically composed of W, Mo, and V. They possess an unusually large size for inorganic molecules and an array of interesting physico-chemical properties, including redox, catalytic, and photochromism [191,192,193]. This work exemplified how the unique photo/redox properties of nanostructured POMs could be translated into a macroscopic material using DLP, as shown Figure 7. Smart materials containing imidazolium-based PILs and photochromic POMs were designed, allowing fine-tuning of the overall properties of the 3D printed material. The beneficial redox properties of the POMs were transferred to the obtained geometrically complex photoactive devices with reversible photochromism. These advanced properties could be useful in different potential applications, such as reversible information storage and temporal reduction/oxidation of pre-formed printed devices [27].

Similarly, recent works have demonstrated that PILs are ideal materials to stabilize perovskite nanocrystals (PS-NCs). Xia et al. recently published a report showing the advantages of protecting hybrid perovskites from decomposition in humid environments with in situ PIL polymerization. The sensitivity to moisture during the cell fabrication was effectively reduced, improving the quality and long-term stability [194]. Nevertheless, PS-NCs are not commonly added as additives into 3D printing materials, because of their instability. Therefore, combinations based on PILs represent a great opportunity for designing photoactive 3D printed devices for photocatalysis, optoelectronic, conductive, electrochemical, and redox applications, among others. 

### 4.2. Antimicrobial and Biomedical

There are many antimicrobial agents that can be added into polymeric matrices. Silver nanoparticles (AgNPs) are commonly used, with several examples of AgNPs-polymeric materials being formulated for additive manufacturing [195,196,197,198]. The nanoparticles (NPs) are either dispersed in the resin before 3D printing or synthesized during the 3D printing process, generating ready-to-use printed devices. However, one problem that appears when mixing neutral polymers with nanoparticles is the aggregation of the NPs and their migration due to a poor stabilization ability [199].

Over the last five years, the use of PILs for antimicrobial applications has expanded considerably. Generally, imidazolium based PILs have been the most widely investigated, due to their outstanding thermal stability, low Tg values, and easy synthesis [200]. The antimicrobial activity of imidazolium salts and their polymer derivatives has been extensively demonstrated [80,201,202,203,204], and in contrast to the cytotoxic ILs, PILs have been shown to be biocompatible [205,206,207,208,209]. 

**Figure 8:**
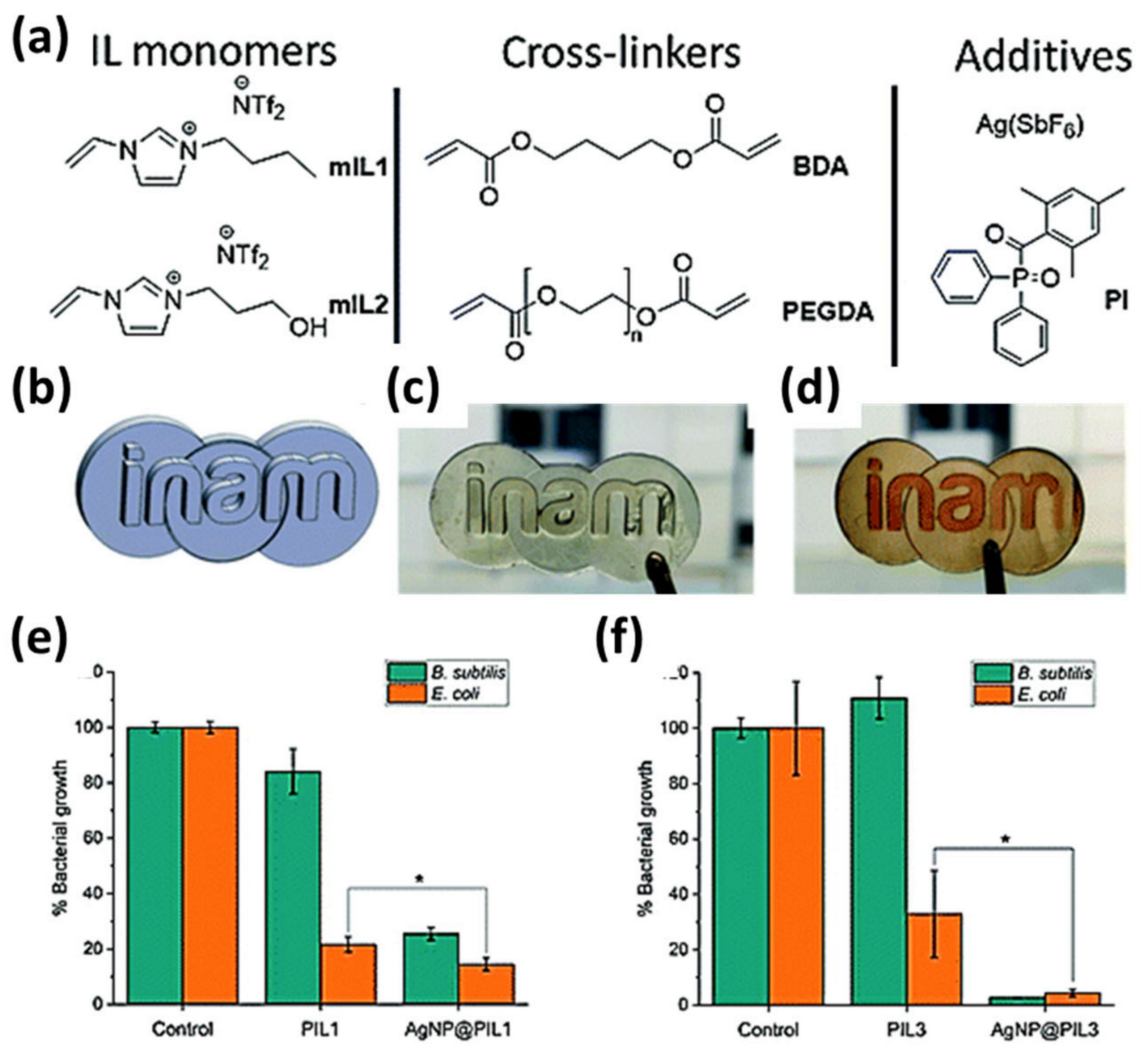
(**a**) The components employed for the development of 3D printable PIL-based formulations; (**b**) CAD design that was employed as a benchmark for 3D-printing; (**c**) 3D-printed part without Ag reduction; (**d**) 3D printed part with AgNPs generated by post printing UV irradiation; (**e**) bacterial cell viability of *E. coli* and *B. subtilis* incubated with PIL1 and AgNP@PIL1; (**f**) bacterial cell viability of *E. coli* and *B. subtilis* incubated with PIL3 and AgNP@PIL3. Reproduced from reference [80] with permission from the Royal Society of Chemistry, under CC BY 3.0 license.

Brushes based on imidazolium PIL with high antibacterial activity have been successfully printed [210]. Furthermore, the inherent ability of PILs to stabilize and support metal precursor salts and nanoparticles during the printing process has been well reported. Sans and co-workers demonstrated the possibility of 3D printing imidazole-based materials with silver salts, while controlling the synthesis and stabilization of silver nanoparticles, as represented in Figure 8. The manufacturing process was successfully decoupled from the generation of silver nanoparticles and the printed films presented an enhanced antimicrobial activity compared to pure PILs [80]. 

**Figure 9:**
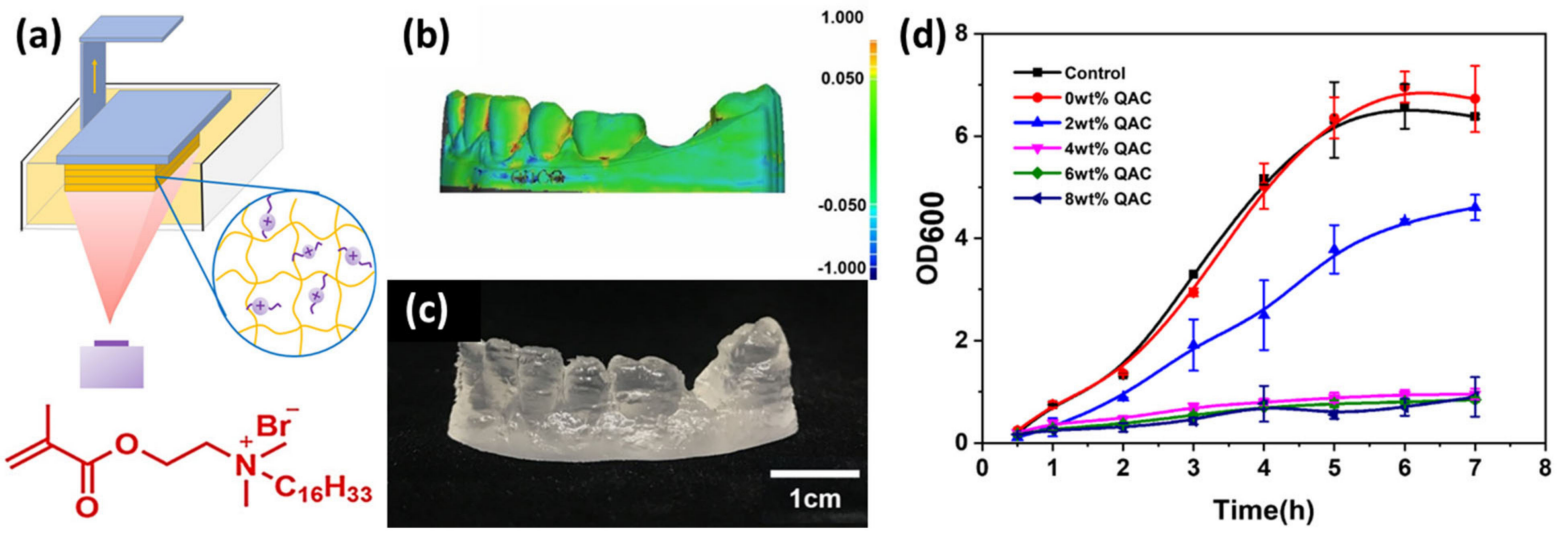
(**a**) Schematic representation of the 3D printing process and the monomeric IL involved in preparing the photosensitive resin; (**b**) picture of the dental model made by DLP 3DP; (**c**) picture of the corresponding scan data; (**d**) growth curves of *E. coli* using PIL formulation with different contents of the antibacterial agent. Adapted from reference [65], with permission.

Apart from imidazole groups, other PIL structures have shown some antimicrobial activity. For example, Li et al. 3D printed a tooth model with high precision DLP, using cholinium-based photopolymerizable ILs with antibacterial properties (Figure 9) [65].

**Figure 10:**
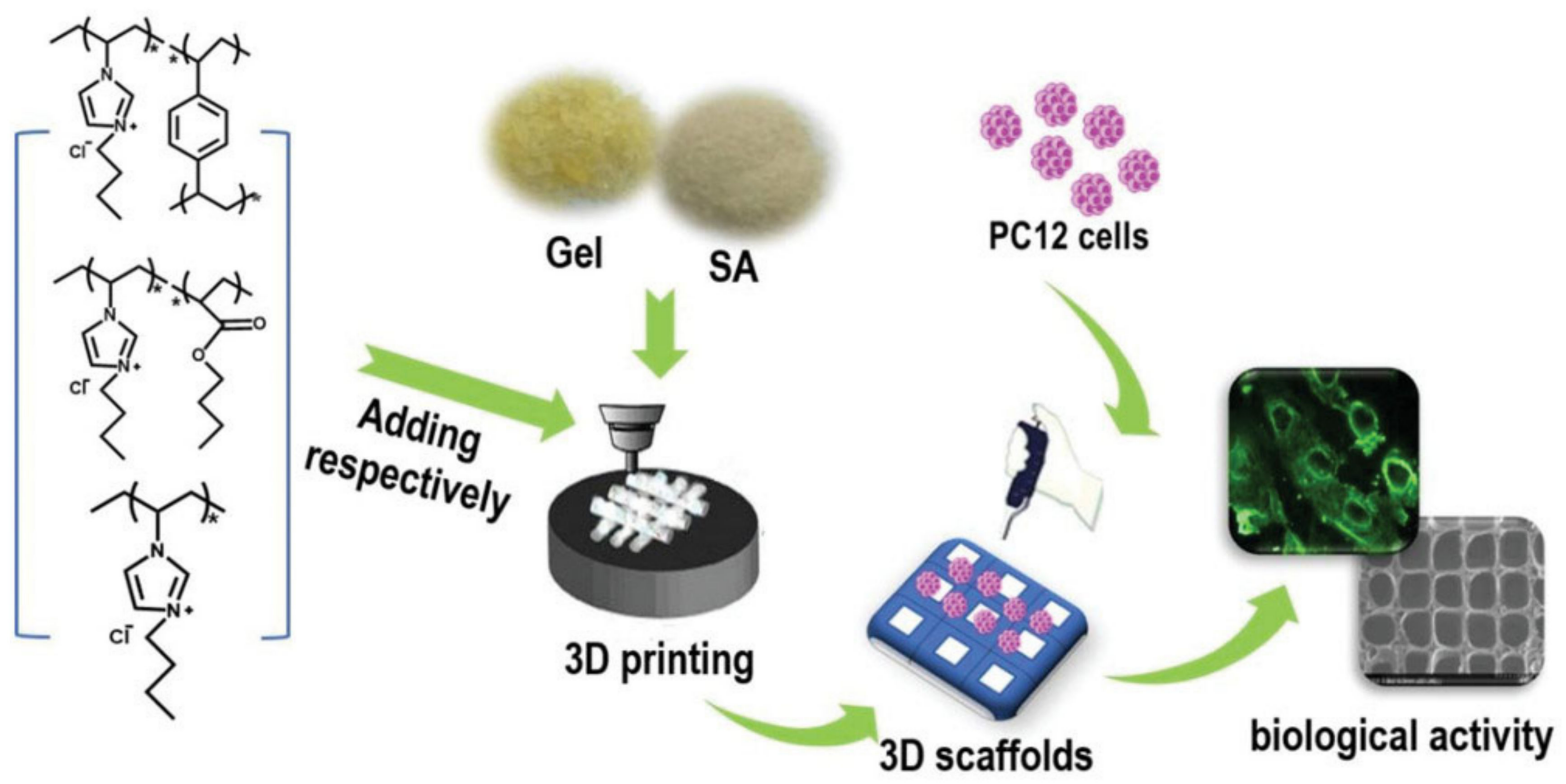
Schematic diagram including the synthesis of PILs, the 3D printing process, and the detection of biological activity. Reproduced from reference [81], with permission.

Antibacterial resins are essential in the biomedical field. Furthermore, AM contributes to the research and development of biomaterials for prototyping complex and customized structures with patient-specific necessities [211]. Bioprinting has become mainstream and ILs and PILs have great potential in this area, owing to their tailorable biocompatibility and antimicrobial properties [212]. In this context, PIL-based bio-inks formulated for 3D printing have been prepared for applications in tissue-engineering (Figure 10) [81].

Additionally, the flexibility to modify the physicochemical properties of these compounds solves most of the issues found in drug delivery systems, such as low bioavailability and drug insolubility [213].

### 4.3. Catalysis

**Figure 11:**
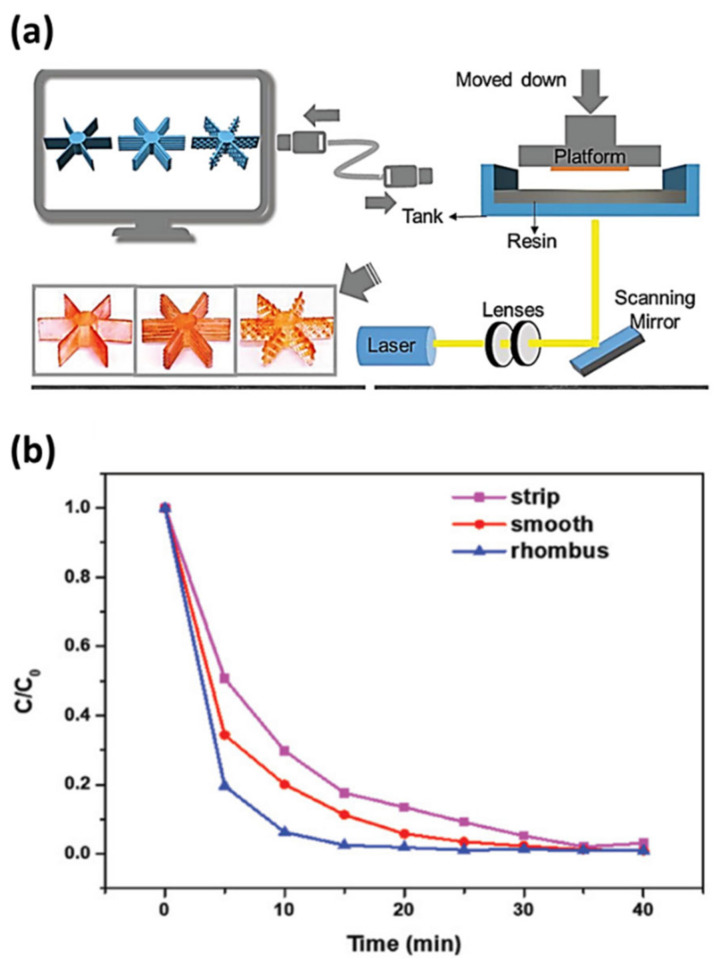
(**a**) Schematic illustration of the agitating impeller preparation from the digital CAD model using SLA 3DP technology; (**b**) degradation curves of 4-nitrophenol (4-NP) catalyzed and stirred by three types of AI–Pd. Adapted from reference [66], with permission.

In catalysis, the use of 3DP is also becoming attractive. The combination of digital control over the geometry, function, and chemical composition of this technique is having a significant effect on preparing reactors and structured catalysts [214]. For instance, 3D-printed magnetic stirrers have been successfully designed and implemented for catalyzing chemical reactions [215,216]. Once the reaction is over, the stirrer can be removed from the reaction media, washed, and reused, without further purification steps. In addition, a 3DP catalytic device based in Cu/Al_2_O_3_ showed a high catalytic efficacy and good recyclability in different Ullmann reactions [217]. Most work in this area have focused on improving mass transfer through geometry optimization. The use of PILs can help in this regard. Wu et al. constructed an agitating impeller (AI), shown in Figure 11, with a prismatic structure and combining 3DP and PILs, and they demonstrated that the mass transfer, catalyst activity, and recovery were enhanced when using printable PILs loaded with palladium ions. Imidazolium PILs with acrylate groups were synthesized and modified on the surface of the impeller through photopolymerization using SLA [66].

In a different example, a cellulose-based catalyst was prepared through AM, using cellulose dissolved in a quaternary-ammonium-based IL. The freedom in design of this manufacturing technique allowed building up thin and homogeneous films of different shapes, types, and designs. As a result, the final catalytic printed parts were implemented in specific reactors for dye removal from wastewater [218].

**Figure 12:**
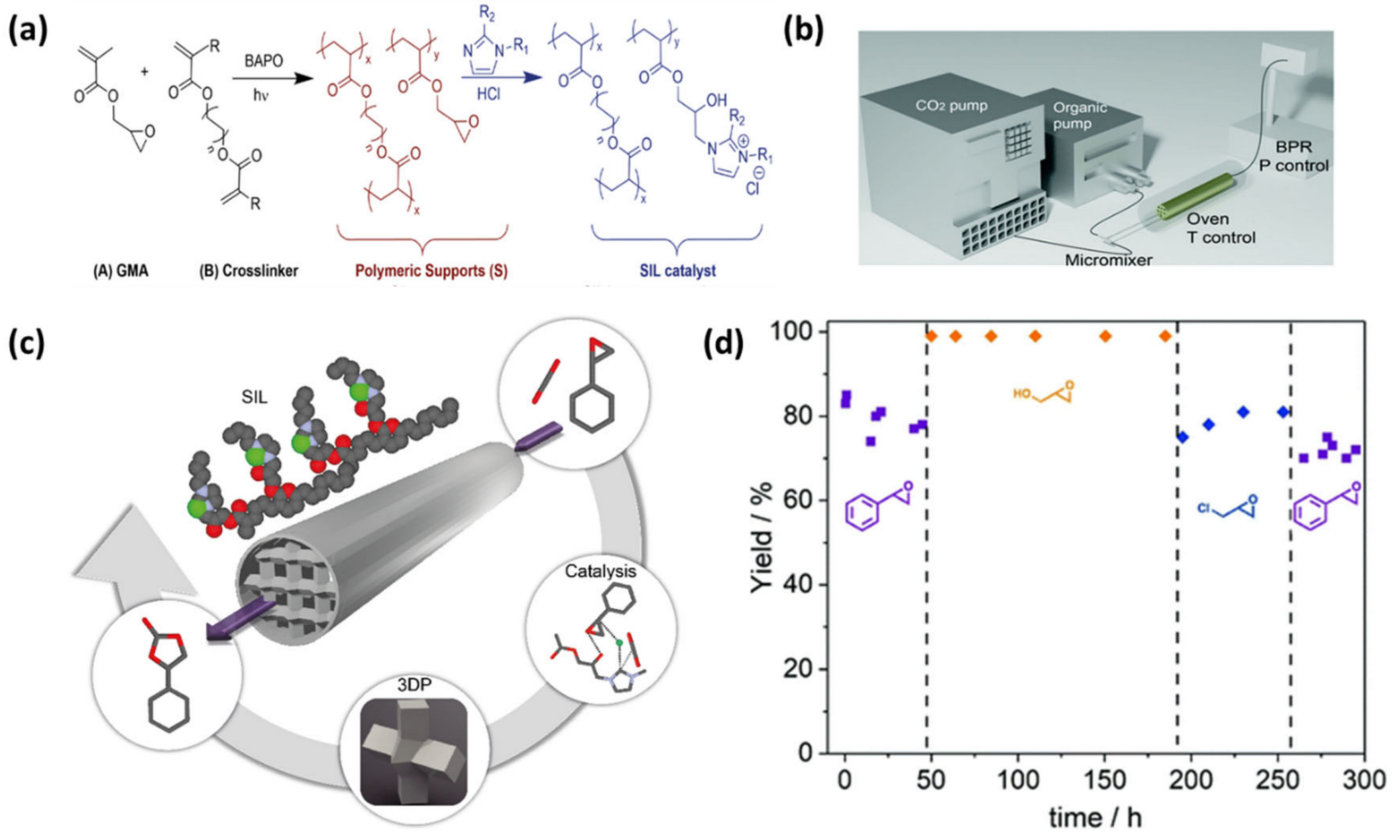
(**a**) Preparation of SILPs; (**b**) continuous flow set-up; (**c**) application of 3DP catalytic reactor column in CO_2_ cycloaddition to epoxide; (**d**) yield vs. time on stream obtained for the continuous flow reaction between different epoxides and CO_2_. Conversion calculated by ^1^H-NMR. Selectivity > 99%. Adapted from reference [220].

Furthermore, microfluidic reactors can be fully 3D-printed with internal flow channels and a high surface quality [219]. The possibility of printing a reactor with a catalytic material properly supported in the channels can be very beneficial for catalyzing reactions. In this regard, our group has reported different 3D printed column architectures using SILPs that were successfully implemented for the catalysis of CO_2_ cycloaddition to epoxide. The formulations were based on the polymerization of glycidyl methacrylate with dimethacrylate as crosslinker, with further functionalization with imidazolium-based ILs (Figure 12a). The catalytic column was used in a continuous-flow process of cyclic carbonate preparation from CO_2_ and epoxide (Figure 12b). The AM generated catalytic reactors with higher catalytic activity than similar sized packed bed reactors. The catalytic activity and stability of the reactor were kept over 300 h, without loss of activity [220].

The introduction of 3DP in catalysis is a growing field, although ILs and PILs have not yet been widely employed. Nevertheless, the use of IL-based materials in catalysis has been extensively studied [221,222,223]. The most common ionic moistures employed in this field are imidazolium cations, although they are quite sensitive to basic conditions. Phosphonium ionic liquids have an intermediate base stability [224,225], while triazolium [226], pyrazolium [227], and cyclic ammonium (such as pyrrolidinium) [228] cations are often more stable in basic conditions, and are therefore more appropriate for the preparation of basic ionic liquids.

### 4.4. Electronics

ILs and PILs, mainly imidazolium-based, have also been widely used for electronic, energy storage, supercapacitor, and conductive applications.

Soft somatosensitive actuators were constructed by direct ink writing with ILs via multi-material embedding, which enables the formation of soft robotic actuators mimicking the human somatosensory system. The sensor ink consisted in a conductive shear-thinning ionogel, formed with an IL and fumed silica as a rheology modifier, and exhibited long-term stability and hysteresis-free performance [79]. 

Extensive research has been devoted to PIL-based electrolytes [22,229,230]; however, their ionic conductivities have resulted too low for practical applications. In this context, as mentioned above, mixing PILs with ILs can drastically enhance their polymeric conductivity. Recently, Darren and co-workers demonstrated that imidazolium PIL/IL-based polyelectrolytes were up to six-times more conductive, as well as more thermally and electrochemically stable, than an analogous neutral polymer. The resulting PIL/IL mixture formed a photopolymerizable resin capable of being printed using the SLA methodology [184].

**Figure 13:**
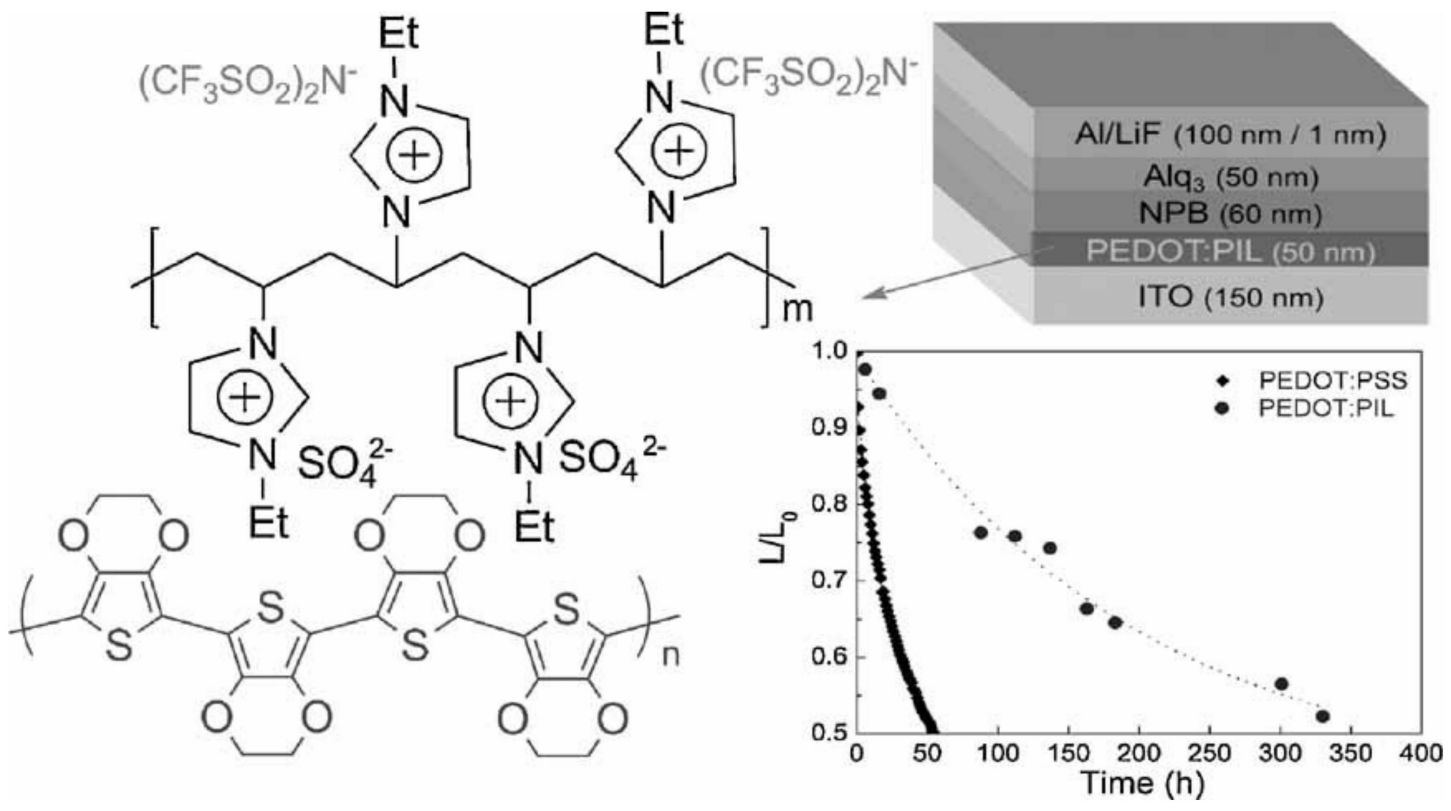
Structure of the fluorescent OLED device, including the chemical structures of PEDOT:PIL and the luminance decay of PEDOT:PSS and PEDOT:PIL devices as a function of time. Reproduced from reference [232], with permission.

On the other hand, combinations of conductive polymers with PILs have also attracted much interest. Conductive polymer dispersions need non-acidic and non-aqueous stabilizers to avoid some issues found during implementation, such as leakage, agglomeration, and solvation. In this regard, new classes of non-volatile organic solvents with hydrophobic nature, such as PILs, have been studied, combining all the beneficial properties of ILs with those of classical polyelectrolytes. Hydrophobic PEDOT/PIL dispersions have been successfully tested by several groups as alternative materials for electrochemical applications, due to their organic nature, large electrochemical window, and thermal stability [22,144,168]. For instance, a fluorescent OLED device was fabricated using PEDOT with an imidazole PIL as a hole-injection layer (Figure 13). In comparison with a control device using a conventional PEDOT:PSS based material, the device with PEDOT:PIL was found to achieve a significant improvement in terms of the device lifetime [231,232].

To the best of our knowledge, to date, there has not been any research involving 3D-printable PEDOT:PIL-based materials. A very promising possibility would be to manufacture all-printed devices. Liquids and colloid gels have been used for this purpose; however, some serious issues appeared when using a strong-acid or a strong-alkaline electrolyte, as this caused damaged to the deposition nozzles. Electrolytes based on ILs are therefore favorable for fully-printed devices, because ILs contribute much less to corrosion and they can work at higher voltages than traditional electrolytes [35].

**Figure 14:**
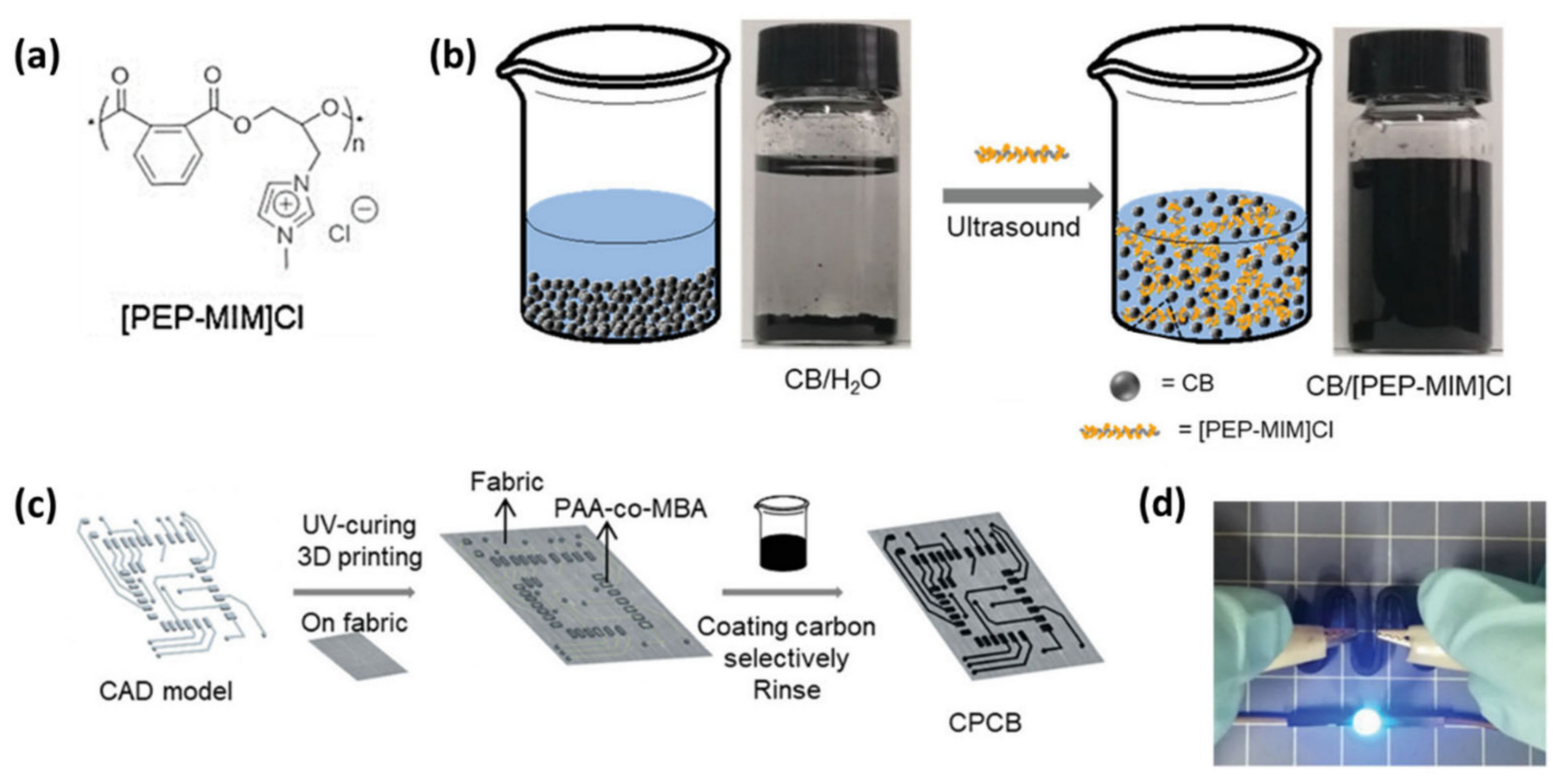
(**a**) Structure of the PIL used; (**b**) dispersion mechanism of carbon black by PIL; (**c**) preparation process starting from the CAD model; (**d**) image of the final device coated with carbon, showing its conductivity. Adapted from reference [26], with permission.

Moreover, PIL materials have been mixed with carbon-based nanocomposites to prepare 3D-printable resins, resulting in very interesting works. In this regard, flexible and wearable conductive polymers were manufactured through vat polymerization, using imidazolium PILs for properly dispersing carbon, as represented in Figure 14 [26]. 

**Figure 15:**
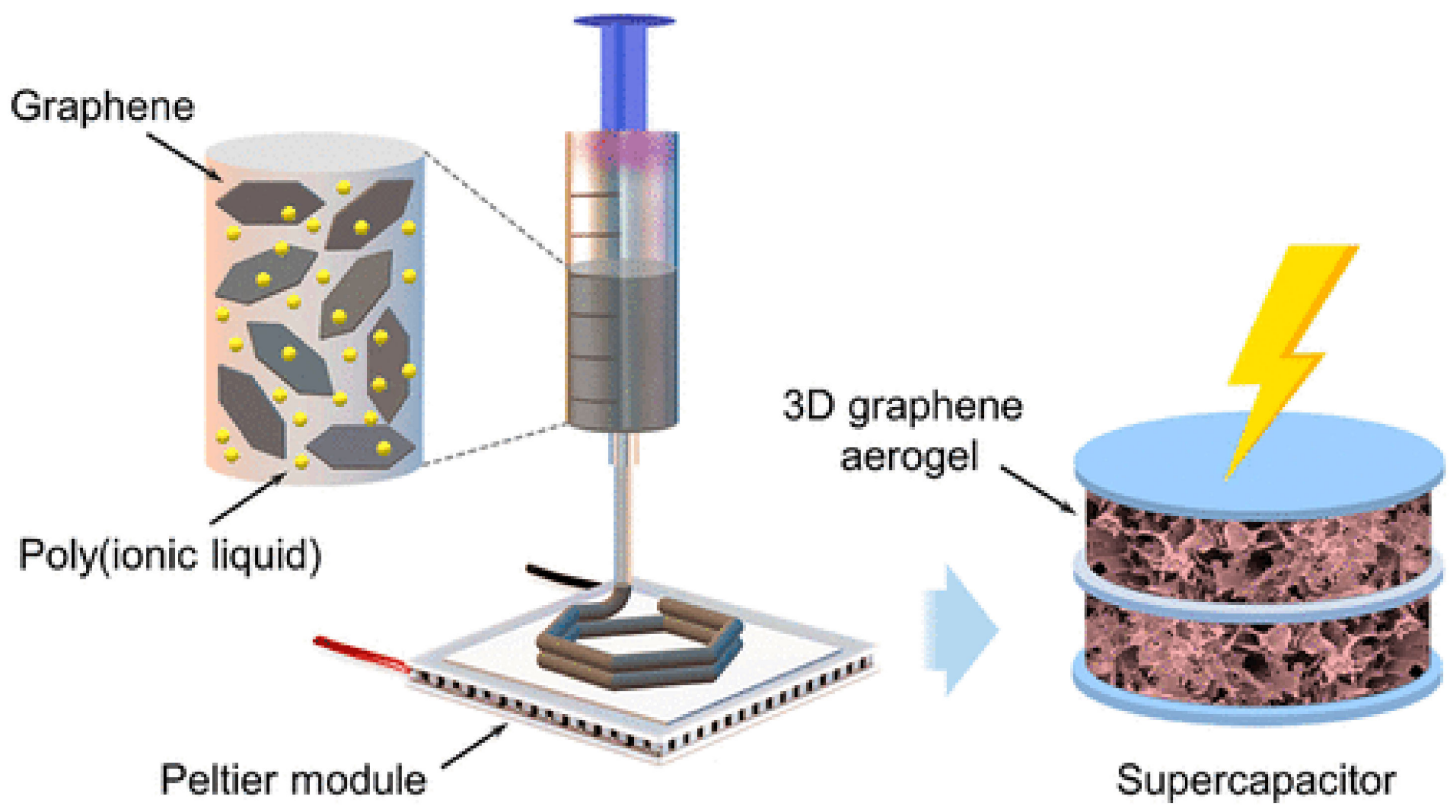
Schematic representation of the PIL-based formulation, the 3D printing process, and the final graphene aerogel. Reprinted with permission from reference [235]. Copyright 2020 American Chemical Society.

Carbon nanotubes (CNTs) have also been 3D-printed with a mixture of ILs and PILs to develop soft electronics, resulting in a significant increase of the composite conductivities (up to 520 Sm^−1^ adding 15 wt% MWCNT) [41,233,234]. Graphene aerogels that can be employed for energy storage applications, such as batteries or supercapacitors, were fabricated using a freeze 3D printing technique, as shown Figure 15. Using an aqueous dispersion of graphene stabilized in imidazolium-based PILs, it was possible to achieve an electrical conductivity of 149 Sm^−1^ [235].

Printable energetic materials based on vinylimidazolium monomers were also photopolymerized using a DLP printer, manufacturing complex geometries at high resolution, while also providing good control over the mechanical and energetic characteristics of the produced objects. The 3D printed polymers allowed reducing the amount of filler, without compromising the final performance of the device [236].

Generally, much progress has been made in printing functional and smart materials, as well as in improving printing technologies. For example, hybrid 3D printing processes that combine different manufacturing processes (multiprocess) have been implemented, resulting in an advanced method to prepare intelligent materials with new and complex functionalities [237]. Even so, there is still a need to find new and more efficient printable materials and, simultaneously, to engineer a printing process capable of introducing multiple materials into a single print, in order to obtain the most from the additive manufacturing.

## 5. Challenges and New Trends in AM

Undoubtedly, 3D printing is a powerful technology that is able to revolutionize industrial processes, from electronic devices to biomaterials. AM technologies have been increasingly researched as innovative techniques for the design and fabrication of advanced parts. Furthermore, they are promising methodologies to obtain environmentally friendly processes [32]. The freedom of design and fabrication that AM offers is leading to the emergence of exciting fields such as 4D printing. Here, the outstanding features of 3DP are combined with stimuli-responsive materials, leading to devices that transform over time in the presence of external stimuli, changing the form or function of 3D objects autonomously [238,239]. As such, 4DP has tremendous potential, promising advancements in existing manufacturing methods, as well as the emergence of new ones in the coming decade [240,241]. Nevertheless, despite all these amazing features, there are still some challenges that require further research and technological development in order to ubiquitously adopt this manufacturing methodology in various industries [242].

One of the main drawbacks is related to the final appearance and mechanical properties of the printed part. For example, the void formation between subsequent layers of materials can be very high, reducing the mechanical performance, due to the additional porosity created by AM. Another common problem is the anisotropic behavior that is caused by the layer-by-layer printing method. The microstructure of the material, as well as the mechanical behavior inside each layer can be different compared to that at the boundaries between layers. The printing direction can also affect the mechanical behavior of nanocomposites from PIL formulations. Not only the internal properties can be affected, but also the device aesthetics can be influenced by the layer-by-layer appearance. Moreover, the finished product can diverge from design to execution, because transferring CAD models into a 3D-printer often results in inaccuracies and defects, particularly in curved surfaces. The generation of supporting materials that are easy to remove after printing, and slicing the part into sufficient layers, as well as post-processing the printed objects are possible solutions to eliminate these defects [29].

Furthermore, there is still a large limitation in the materials suitable for 3D printing. The development of printable inks and processable filaments is crucial to improve the production process and pattern definition. The materials and their qualities are important, not only for the final application, but also for the manufacturing process. Not all the photoactive monomers can be properly photopolymerized under printing conditions. The desirable thermal and mechanical properties can be achieved, functionalizing the monomeric ILs, which may also modify the polymerization kinetics. Likewise, photo-initiators can also affect the degree of polymerization and the final properties of devices. Apart from the main components of the photopolymer resin (monomers and photo-initiator), various additives can be mixed into the formulation. The combination of multiple compounds must be compatible and homogeneously distributed during printing, to produce effective devices with high resolution and without altering the final properties. 

Another important challenge is the optimization of printable smart materials to fabricate entire smart systems. Most of the AM processes can only manufacture one or two components of a device, and not the full device. Thus, a one-step printing process of a whole device is a promising route to develop fully-integrated usable devices [34]. Electrolytes based on ILs are very favorable for fully-printed devices, because ILs contribute much less to corrosion and they can work at higher voltages than traditional electrolytes [35]. In order to solve these technological issues, the combination of different 3DP techniques [243], as well as combining emerging 3D printing processes with traditional fabrication techniques, would be beneficial. 

In addition, modern society demands other requirements related to sustainable and economical procedures. As a result, we suggest that new trends to improve 3D printing will mainly focus on:

**1. Technological evolution**: Developing more user-friendly software and printers, which must be accessible to a broad range of users and employ open-source coding. In addition, the printing speed and 3D scanning need to be improved in the context of mass production. Moreover, the final appearance and the mechanical performance must enhanced, avoiding the layer-by-layer issues. Investment and research are required to reduce prices and increase quality. This is the only way that these technological advances will arrive to all businesses, as well as the general public [244].

**2. Materials**: Finding novel and cheap PILs to meet the growing demand for multi-material smart prints. Not only are technological advancements required to enhance the printing speed, consistency, and quality of printed goods, but the materials used are also critical. Tailor-made photopolymers can be employed by 3DP technologies to achieve specific features. The fusion of AM with thermal and electro-responsive PILs, allowing 4D printing, is a revolutionary field, with enormous potential in the future. All of the components involved, however, must be compatible and optimized for the manufacturing process. Therefore, intensive research in the structure–property relationships of ILs and PILs is essential to discover innovative resins suitable for 3DP, to compete with established procedures and unleash new applications [245].

**3. Sustainability**: Investing in biodegradable and bio-sourced PIL-based materials. Developing advanced materials based on products from renewable resources that are sustainable, non-cytotoxic, and that comply with circular economy concepts is therefore imperative, to drive innovation and take full advantage of the growth of 3DP. In addition, this technology can be employed for applications related to renewable energy and energy efficiency, and is able to recycle waste plastics as printable materials. Hence, there are many opportunities to exploit in this area. Moreover, manufacturing goods in the same place where they will be used will promote local production and will reduce shipping and environmental costs. These are some of the requirements being currently explored for Industry 4.0 [246,247].

**4. Affordability**: Lowering the costs of 3D printing equipment, materials, and post-printing processes, in order to expand the usage of this technology worldwide. As a result of advances in engineering techniques and optimization of materials, a simultaneous reduction in operating costs will be achieved [60].

As discussed, many of the materials used in AM are not yet optimized for the fabrication process. Polymeric ILs are appearing and represent a very desirable material, capable of properly stabilizing nano and molecular structures. In a short period of time, printable PILs have had a notably impact across several sectors of 3D printing, expanding the uses of this discipline and opening up alternative options in material design. To date, several researchers in the 3DP world have used ILs and PILs, and we forecast that their use will continue to grow. However, in order to overcome the limitations imposed by AM, more sophisticated ionic liquids must be created, taking advantage of their tunability. It is imperative to study the synergic interactions between the components that form these novel materials, to develop effective photopolymer resins. Undoubtedly, learning more about the factors that influence the obtainment of the composites would aid in the design of more efficient synthesis routes, which would improve the performance of the final material and devices.

## 6. Conclusions

In summary, the evolution of additive manufacturing in recent years has been phenomenal. The increased funding, research, and development worldwide will result in a fast transition from conventional fabrication to 3DP manufacturing, both at laboratory- and large-scale. This technology also allows us to go a step further, by enabling 4D printing, and it will a key factor in the success of the Forth Industrial Revolution 4.0. However, there are still several issues that need to be resolved in order to compete with traditional methods in bulk production. Hence, intensive research is essential to get the most out of 3DP.

In this review, we have outlined the major progress and efforts made in the development of 3D printable advanced materials. In particular, the advancements and benefits of stabilizing smart composites into PILs set the stage for next-generation devices. 

## Figures and Tables

**Table 1:** Overview of AM classification [39].

Process Principle	AM Technology	Materials	Features
Vat photopolymerization (VP)	Stereolithography (SLA) Dynamic light processing (DLP) Continuous Liquid Interface Production (CLIP)	Photopolymer Ceramic	High resolution Slow process High cost
Powder bed fusion (PBF)	Selective laser sintering (SLS) Selective laser melting (SLM) Electron beam melting (EBM)	Metal Polymer Ceramic	High resolution Slow process High cost
Material extrusion	Fused filament fabrication (FFF) Direct ink writing (DIW)	Polymer Ceramic Biomaterials	Low resolution Fast process Low cost
Material Jetting (MJ)	Ink-jetting Thermojet Polyjet	Photopolymer Wax	High resolution Fast process
Binder Jetting (BJ)	Ink-jetting	Metal Polymer Ceramic	High resolution Slow process High cost
Direct Energy Deposition (DED)	Direct Metal Deposition (DMD) Laser Deposition Laser Consolidation	Metal Powder Wire	Low resolution Fast process Low cost
Sheet lamination (SL)	Ultrasonic consolidation Laminated object manufacturing (LOM)	Hybrids Metallic Ceramic	Low resolution Fast process Low cost
